# Oncogenic driver and therapeutic target: Prolactin signalling axis in retroperitoneal sarcoma

**DOI:** 10.1002/ctm2.70669

**Published:** 2026-05-05

**Authors:** Fu'an Xie, Lingwei Gu, Kunrong Yang, Shuai Wang, Mengmeng Xiao, Guangting Yan, Quan Zhang, Rubing Liang, Aobo Zhuang, Zhe Xi, Yujia Niu, Yanhua Chen, Xiaogang Xia, Linlin Qu, Bin Zhao, Weibing Li, Ting Wu, Chundong Yu, Chenghua Luo, Houzhao Wang, Lanlan Lian, Wengang Li

**Affiliations:** ^1^ Cancer Research Center Xiang'an Hospital of Xiamen University, School of Medicine, Xiamen University Xiamen Fujian China; ^2^ National Institute for Data Science in Health and Medicine Xiamen University Xiamen Fujian China; ^3^ Xiamen University Research Center of Retroperitoneal Tumor Committee of Oncology Society of Chinese Medical Association, Xiamen University Xiamen Fujian China; ^4^ Xiamen Treatgut Biotechnology Co., Ltd. Xiamen Fujian China; ^5^ Laboratory of Biochemistry and Molecular Biology Research Department of Clinical Laboratory, Clinical Oncology School of Fujian Medical University, Fujian Cancer Hosptial Fuzhou Fujian China; ^6^ Department of Cardiology Xiamen Key Laboratory of Cardiac Electrophysiology, Xiamen Institute of Cardiovascular Diseases The First Affiliated Hospital of Xiamen University, School of Medicine, Xiamen University Xiamen Fujian China; ^7^ Department of Retroperitoneal Tumor Surgery Peking University People's Hospital Beijing China; ^8^ State Key Laboratory of Celluar Stress Biology Faculty of Medicine and Life Science, Xiamen University Xiamen, Fujian China; ^9^ Department of Laboratory Medicine Fuqing Maternal And Child Health Care Hospital Fujian China; ^10^ Department of Hepatobiliary Surgery Xiang'an Hospital of Xiamen University, School of Medicine, Xiamen University Xiamen Fujian China; ^11^ Department of Laboratory Medicine Xiang'an Hospital of Xiamen University, Xiamen University Xiamen Fujian China; ^12^ Department of Laboratory Medicine Dongfang Hospital, Xiamen University Fuzhou Fujian China

**Keywords:** c‐MYC, hyperprolactinaemia, lactose, prolactin, retroperitoneal sarcoma, SOX4

## Abstract

**Background:**

Retroperitoneal sarcoma (RPS) is a type of malignant tumour arising from mesenchymal tissues within the retroperitoneal space. RPSs tend to develop covertly and are often undiscovered when they have already grown significantly and invaded surrounding tissues and organs. These malignancies demonstrate high recurrence rates, present surgical challenges and exhibit limited responsiveness to radiotherapy and chemotherapy. Serum‐derived molecules are known to play critical roles in tumourigenesis and tumour progression. However, the serum molecular profile of RPS patients remains unclear.

**Methods:**

We performed multi‐omics analysis of serum samples from patients with retroperitoneal dedifferentiated liposarcoma. Prolactin concentrations were quantified using Enzyme‐Linked Immunosorbent Assay (ELISA). RNA‐seq facilitated the identification of candidate signalling pathways, while gene expression was validated through quantitative polymerase chain reaction, immunohistochemistry and western blot analyses. Molecular mechanisms underlying transcriptional regulation were investigated through Chromatin Immunoprecipitation‐qPCR (ChIP‐qPCR) and dual‐luciferase reporter gene assays.

**Results:**

Integrative multi‐omics profiling identified significant perturbations in galactose metabolism coupled with marked elevation of prolactin (PRL) levels in Retroperitoneal Liposarcoma (RLPS) patients. Further screening of serum prolactin levels in 100 patients with retroperitoneal tumours revealed that 90% of the cases exhibited hyperprolactinaemia in our research cohort, encompassing both malignant sarcomas and benign tumours. Studies at the clinical sample, cellular and animal levels have found that abnormally elevated prolactin in the serum can originate from sarcoma tissues. Mechanistic investigations identified SRY‐box transcription factor 4 (SOX4) as a previously unrecognised transcriptional regulator of PRL. Functionally, PRL not only enhanced liposarcoma cell and fibrosarcoma cell proliferation but also conferred resistance to MDM2 inhibitors. Signalling pathway analysis revealed that PRL activates the Janus Kinase–Signal Transducer and Activator of Transcription Pathway (JAK–STAT) signalling pathway and up‐regulates c‐MYC expression.

**Conclusions:**

This study indicates that PRL can serve as an oncogenic driver and therapeutic target. The identification of SOX4–PRL–c‐MYC signalling axis provides actionable insights for developing novel therapeutic strategies against this malignancy.

**Key points:**

- Retroperitoneal sarcoma cells can secrete prolactin into the bloodstream, inducing hyperprolactinaemia, which subsequently triggers metabolic reprogramming, such as glucose metabolism.
- SOX4 can function as a transcription factor that facilitates PRL transcription.
- PRL can activate the JAK–STAT signalling pathway by binding to PRLR on sarcoma cells, leading to the up‐regulation of c‐MYC.

## Graphical abstract

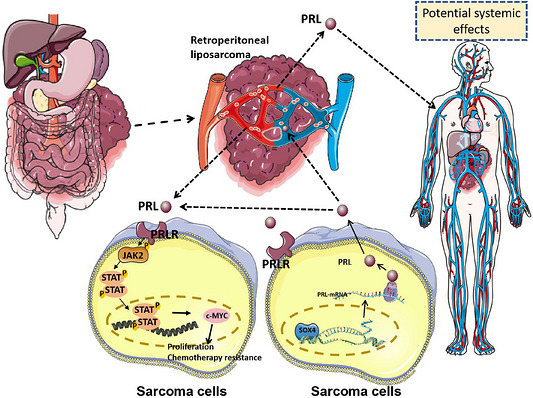

## 1 INTRODUCTION

Sarcomas constitute a rare class of neoplasms derived from mesenchymal origin, encompassing malignancies arising from diverse tissues including adipose, fibrous, neural, muscular and vascular tissues. These heterogeneous tumours demonstrate extensive biological diversity, with over 100 histologically distinct subtypes distributed across multiple anatomical compartments: retroperitoneal space, trunk, head and neck regions, visceral organs and extremities. The current World Health Organization classification system stratifies sarcomas into three principal categories: (1) soft tissue sarcoma (STS), (2) visceral sarcoma and (3) bone sarcoma, reflecting their distinct biological behaviours and therapeutic implications.^1^, ^2^ STS accounts for 1% of adult solid tumours.^3^

Due to its unique anatomical location, sarcomas in the retroperitoneal space tend to develop insidiously. Retroperitoneal sarcomas (RPSs) frequently present as large masses that simultaneously invade and encase multiple adjacent organs, demonstrating greater biological aggressiveness compared with extremity sarcomas.^4^ The clinical management of RPS presents distinct challenges, marked by heightened surgical complexity, elevated recurrence rates and diminished chemosensitivity. In addition, some benign and metastatic tumours often occur in the retroperitoneum. They, along with RPSs, are collectively referred to as retroperitoneal tumours. While emerging molecular insights have provided new therapeutic targets, pharmacotherapeutic development for RPS remains in its infancy. Consequently, comprehensive investigation into its molecular pathogenesis is essential for developing novel therapeutic strategies aimed at improving survival outcomes and quality of life.^5^ With RPS receiving increasing attention, more and more hospitals or medical organisations have established specialised retroperitoneal tumour surgery departments, including the Transatlantic Australasian RetroPeritoneal Sarcoma Working Group (TARPSWG) and the Retroperitoneal Tumor Group of the Chinese Medical Association.

Despite substantial research efforts dedicated to identifying therapeutic targets and diagnostic biomarkers for retroperitoneal tumours, significant clinical progress remains limited. Accumulating evidence highlights that serum‐based components – including hormones, proteins, metabolites and non‐coding RNAs – critically regulate oncogenic processes by directly or indirectly modulating tumour cell proliferation, apoptosis, metastasis and immune escape. Importantly, dynamic fluctuations in the serum concentrations of these molecules hold substantial potential as non‐invasive biomarkers for early tumour detection, prognostic stratification and therapeutic response monitoring. For instance, PRL promotes oncogenesis and disease progression in multiple malignancies through autocrine/paracrine mechanisms, with tumourigenic effects observed in breast carcinoma, ovarian cancer, prostate adenocarcinoma and hepatocellular carcinoma.^6^, ^7^, ^8^

PRL is a 199‐amino acid polypeptide hormone primarily synthesised and secreted by lactotrophs in the anterior pituitary gland. The physiological functions of PRL are multifunctional, encompassing: mammary gland development, lactogenesis, maintenance of secretory epithelium, testosterone biosynthesis and accessory gland development. The physiological range of serum PRL exhibits interindividual variability, with basal concentrations typically <20 ng/mL. Hyperprolactinaemia is generally defined as PRL levels exceeding 20 ng/mL (424 mIU/L, males) or 25 ng/mL (530 mIU/L, females).^9^

To identify novel serum biomarkers and therapeutic targets, we performed comprehensive multi‐omics analyses, including targeted metabolomics, untargeted metabolomics, lipidomics and proteomics, on matched serum samples from 10 retroperitoneal dedifferentiated liposarcoma (RDDLPS) patients and 10 healthy controls. We observed a significant dysregulation of galactose metabolism and elevated circulating PRL levels. This investigation aims to elucidate the molecular mechanisms underlying metabolic reprogramming and endocrine dysregulation in PRS, as well as characterise PRL's functional role in RPS pathogenesis through in vitro and in vivo models and explore therapeutic potential via prolactin–prolactin receptor (PRLR) signal axis.

## 2 MATERIALS AND METHODS

### 2.1 Study population

In terms of clinical serum samples, the number of samples is as follows: healthy subjects (*n* = 169), parturient women (*n* = 100), lactating women (*n* = 17), kidney transplant recipients (*n* = 37), breast cancer patients (*n* = 48), hyperprolactinaemia cases (*n* = 100), adrenal mass patients (*n* = 12), malignant melanoma patients (*n* = 31), breast mass patients (*n* = 304), liver cancer patients (*n* = 28), lung cancer patients (*n* = 13), pituitary tumour patients (*n* = 34), gastric cancer patients (*n* = 65), oesophagus cancer patients (*n* = 16), colorectal cancer patients (*n* = 35), prostate cancer patients (*n* = 2), uterine leiomyoma patients (*n* = 5), malignant tumour of uterine body patients (*n* = 7), endometrial cancer patients (*n* = 11), cervical cancer patients (*n* = 45) and retroperitoneal tumours patients (*n* = 100). The inclusion criteria for the preoperative group were patients who had not taken opioids or chemotherapy drugs and had not undergone surgery when they came to the hospital. The inclusion criteria for the post‐operative group were patients who had stopped taking opioids 1 week after surgery and had not taken chemotherapy drugs. Blood samples were collected on an empty stomach at around 7 a.m. In terms of clinical tumour tissue specimens, the number of specimens is as follows: colorectal cancer patients (*n* = 8), liver cancer patients (*n* = 8), paracancerous adipose tissue (*n* = 30) and retroperitoneal liposarcoma (*n* = 100). We collected matched pairs of frozen and fixed normal and tumour samples from 100 patients with RPS. All samples were collected from Xiang'an Hospital of Xiamen University and Peking University International Hospital. Fixed samples were transferred to Shanghai Outdo Biotech Co., Ltd. for tissue ChIP assays, while the frozen samples were prepared for proteomic, metabolic and lipidomic analyses. The present protocols were reviewed and approved by the Ethics Committees of all participating institutions, including Xiang'an Hospital of Xiamen University (No. XAHLL2021024, XAHLL2023004), Peking University People's Hospital (No. 2024PHB240‐001) and Peking University International Hospital (WA2020RW29). All participants were enrolled and anonymised after approval by the institutional review board. Written informed consent was obtained from all participants except those who could not be contacted due to a lack of follow‐up. In these cases, permission was granted by the institutional review boards at each participating institution for the use of existing tissue samples for research.

### 2.2 Mice

The SOX4 gene knockout mice of adipose tissue were provided by Prof. Boan Li at Xiamen University. The detailed production methods were described in their previous reports.^10^ The 4‐ to 6‐week‐old BALB/c (C57BL6) mice were purchased from the SLRC Laboratory Company and kept in a specific‐pathogen‐free environment at the Laboratory Animal Center, Xiamen University (Xiamen, China). They were housed in plastic cages and maintained in a climate‐controlled animal room with a 12/12 h light/dark cycle at 23 ± 1°C and 55 ± 5% humidity. Food and water were provided ad libitum. For the xenograft experiment cells were detached and resuspended in serum‐free medium. Subsequently, 5.0 × 10^6^ cells were injected retroperitoneally or subcutaneously into each mouse using a volume of 100 µL (*n* ≥ 4/group). The humane endpoint was set when any mouse's tumour diameter exceeded 20 mm, at which point the experiment was terminated. All mice were euthanised by CO_2_ exposure at a flow rate of 1.2 L/min, which displaced 20% of the cage volume per minute. Death was confirmed by persistent unconsciousness and absence of breathing. The resulting tumours were completely dissected and weighed. The protocols for the xenograft and metastasis experiments were approved by the Animal Ethics Committee of Xiamen University (approval No. XMULAC20210080).

### 2.3 LC–MS/MS for targeted metabolomics

The LC–MS analysis was performed using an Exion LC system (AB SCIEX) with a ZIC‐pHILIC column (100 mm × 2.1 mm, Millipore) connected to a QTRAP‐5500 mass spectrometer (AB SCIEX). For the LC conditions, 2 µL samples were injected and analysed. The flow rate was set at .2 mL/min. The column and tray temperatures were set at 40 and 4°C, respectively. Mobile phase A contained 15 mmol/L ammonium acetate and 3 mL/L ammonium hydroxide (>28%) in water, while mobile phase B was a solution of acetonitrile in water with a concentration of 90%. The gradient elution program was as follows: maintaining 95% mobile phase B for 1 min, decreasing to 45% mobile phase B over 14 min and holding for 2 min, then increasing to 95% mobile phase B within half a minute and holding for four and a half minutes. In negative ion MRM mode, the ESI voltage was set to −4500 V; in positive MRM mode, it was set to +5500 V. The ion temperature was maintained at 500°C and the curtain gas flow rate at 35 µL/min. The LC–MS/MS conditions were controlled by Analyst software version 1.7.1, and the final data were processed by Multi‐quant software version 3.0.3.

### 2.4 LC–MS/MS for lipidomics

The supernatant of the dried lipid dissolved in the mobile phase was injected into a Thermo Accucore™C30 (2.6 µm, 2.1 mm × 100 mm) column. The column temperature, flow rate and injection volume were set to 45°C, .35 mL/min and 2 µL, respectively. The mobile phase consisted of acetonitrile: water (60:40, v/v) containing .1% formic acid and 10 mmol/L ammonium formate (A) and acetonitrile: isopropanol (10:90, v/v) containing 0.1% formic acid and 10 mmol/L ammonium formate (B). The gradient program initiated from A:B (80:20, v/v) at 0 min, A:B (70:30, v/v) at 2.0 min, A:B (40:60, v/v) at 4 min, A:B (15:85, v/v) at 9 min, A:B (10:90, v/v) at 14 min, A:B (5:95, v/v) at 15.5 min, held for 1.8 min and returned to A:B (80:20, v/v) at 20 min. Mass spectra were acquired on a SCIEX triple quadrupole‐linear ion trap mass spectrometer (QTRAP) 6500 + LC–MS/MS system, equipped with an ESI turbo ion spray interface, operating in positive and negative ion modes and controlled by Analyst 1.6.3 software (SCIEX). The ESI source temperature and ion spray voltage was set at 500°C and 5500 V (positive) and −4500 V (negative), respectively. The ion source gas I, gas II and curtain gas were set at 45, 55 and 35 psi, respectively. Metabolomic and lipidomic data were imported into the online software MetaboAnalyst (https://www.metaboanalyst.ca/) for multivariate statistical analysis. The number of biological replicates in each experiment is listed in the figure legends. Statistical analyses of LC–MS data are described in the figure legends. Except where indicated, statistically significant differences were assessed by a two‐tailed paired *t*‐test.

### 2.5 Tandem mass tag based proteomic analysis

Serum proteomic analysis was conducted in collaboration with the commercial protein analytics provider Jingjie Biotechnology Co., Ltd. (Hangzhou, China). The analysis employed isobaric tags for relative and absolute quantitation and tandem mass tags methodologies on mass spectrometry platforms from AB Sciex and Thermo Fisher Scientific. Principal instrumentation included the Bruker timsTOF Pro, Thermo Scientific Orbitrap Exploris 480, Thermo Scientific Q Exactive HF‐X, Thermo Scientific Orbitrap Fusion Lumos, Thermo Scientific Q Exactive Plus, Thermo Scientific Orbitrap Fusion and Thermo Scientific Q Exactive (http://www.ptm‐biolab.com.cn/serverDetail.html?id=15&serviceCateId=23).

### 2.6 Gene set enrichment analysis

Gene set enrichment analysis (GSEA) was used to identify gene sets and pathways associated with the gene expression data. The enrichment of the Kyoto Encyclopedia of Genes and Genomes (KEGG) in the expression data was assessed using GSEA (http://www.broadinstitute.org/gsea/index.jsp). The results were visualised using the ggplot2 and ClusterProfiler packages.

### 2.7 Enzyme‐linked immunosorbent assay

The concentration of PRL was determined using ELISA kit of PRL (ml058210, ml001906; mlBio, Shanghai, China) according to the manufacturer's instructions.

### 2.8 Cell lines and regents

The SW872 (HTB‐92) and HT1080 (CCL‐121) cell lines were purchased from ATCC. The WEHI164 (IM‐M110), MSC (IMP‐H122), HUVEC (IMP‐H031), BEAS2B (IM‐H128), 293T (IM‐H222), HEEC (IM‐H467), CACO2 (IM‐H092), MCF7 (IMD‐001), A549 (IM‐H113), HUH7 (IM‐H040), QBC939 (IM‐H226), HCT116 (IM‐H098), KYSE30 (IM‐H311), HEC‐1‐A (IM‐H021), Ishikawa (IM‐H018), 3T3L1 (IM‐M045), 93T449 (IM‐H403) and 94T778 (IM‐H482) cell lines were purchased from Xiamen Immocell Biotechnology Co., Ltd. Adipocytes are induced to undergo adipogenic differentiation by mesenchymal stem cells (MSCs) utilizing an adipogenic differentiation^11^ kit (IMC‐012; Immocell, Xiamen, China). Adipocyte1, Adipocyte2, XMU‐RC‐1 and XMU‐RC‐2 were isolated by our team from clinically derived dedifferentiated liposarcoma and adjacent adipose tissues. Cells were cultured in DMEM (IMC‐201; Xiamen Immocell Biotechnology Co., Ltd.) containing penicillin and streptomycin (IMC‐601; Xiamen Immocell Biotechnology Co., Ltd.) and 10% foetal bovine serum (FBS; P30‐3302; PAN). Recombinant Human Prolactin Protein (10275‐H08B) was purchased from Sino Biological. Bromocriptine (T26550A, national medicine permission number: HJ20160170) was purchased from GEDEON RICHTER. Levodopa tablets (19230303, national medicine permission number: H31020888) were purchased from Shanghai Fuda Pharmaceutical Co., Ltd. Cisplatin (HY‐17394), doxorubicin (HY‐15142A), gemcitabine (HY‐17026), RG7112 (HY10959), Nutlin‐3a (HY10029), SAR405838 (HY18986), SP141 (HY141), 10058‐F4 (HY‐12702) and abemaciclib (HY16297A) were purchased from MedChemExpress LLC. Antibodies were purchased from the following sources: PRLR (A4121; ABclonal, China), c‐MYC (A19032; ABclonal), c‐MYC (GB13076‐50; Servicebio, China), PRL (GB14138‐50; Servicebio), p‐STAT3 (AP0705; ABclonal), p‐STAT3 (AP0758; ABclonal), STAT3 (9139s; CST, USA), STAT3 (A21228; ABclonal) and GAPDH (TA08; ZSGB‐BIO, China). Human DA ELISA kit (ml823681L), mouse DA ELISA kit (ml002024L) and mouse PRL ELISA kit (ml001906S) were purchased from Shanghai Enzyme‐linked Biotechnology Co., Ltd.

### 2.9 Gene editing and knockdown

The sgRNA was cloned into the CRISPR‐V2 vector (addgene; #52961) as previously described^12^ in order to generate CRISPR knockout cells. HEK293T cells were transfected with LentiCRISPRv2–sgPRLR or LentiCRISPRv2–sgPRL, along with psPAX.2 and pMD2.G lentiviral packaging system using lipofectamine 2000 reagent (Life Technologies), following the manufacturer's instructions. After 72 h, lentivirus particles in the medium were collected and filtered, then used to infect SW872 and HT1080 cell lines. Puromycin was added to obtain puromycin resistance non‐clonal pool cell lines, the gRNA sequences are provided in Table S7. The human overexpressed plasmids of SOX4 (pLVML–FLAG–SOX4–IRES–puro and pLVX–HA–IRESZSgrenn–SOX4) and the knockout plasmids of SOX4 (pL–CRISPR–EFS–GFP–SOX4–knockout) were provided by Prof. Shuangbo Kong at Xiamen University.^13^ The mouse overexpressed plasmid of SOX4 (pLV–SOX4–puro) and the mouse knockout plasmid of SOX4 were provided by Prof. Boan Li at Xiamen University.^14^ To achieve knockdown of the MDM2 gene, the knockdown primers were inserted into the pll3.7‐neo vector, which was then packaged into viral particles and used to infect target cells. Transduced cells were selected using G418, and the specific knockdown primer sequences are provided in Table S7.

### 2.10 ChIP‐qPCR

The 293T cells were transfected with pLVML–FLAG–SOX4–IRES–puro plasmid, and the transfection was allowed to proceed for 48 h. For fixation, the cells were treated with 1%PFA at 37°C for 15 min in 100 mm cell culture dish. To stop the fixation process, .125 M glycine was added and incubated for 5 min at room temperature. Then, the cells were washed with cold PBS with 1 mM PMSF and lysed with ChIP lysis buffer (50 mM Tris–HCl, pH 8.1, 10 mM EDTA, 1% SDS) containing protease inhibitor and phosphatase inhibitors for 15 min on ice and an ultrasonic processor (SCIENTZ; Cat#SCIENTZ08‐IIIA) was used on ice. Subsequent steps were performed according to the biotechnology protocol of ChIP Assay Kit (P2078; Beyotime, Shanghai, China), and the enrichment of DNA was measured using qPCR and normalised with the input sample. The primer sequences can be found in Table S7.

### 2.11 Dual‐luciferase reporter assay

Construction of luciferase reporter was performed as previously described.^15^ The promoter regions of PRL were amplified from genomic DNA and subcloned into pGL3 plasmid. All constructs were transiently transfected into 293T cells using Lipofectamine 2000. Total cell lysates were prepared 36 h after transfection, and luciferase activity was measured using the Dual‐Luciferase Reporter Assay System (Promega Corporation). Firefly luciferase activity was normalised by Renilla luciferase activity.

### 2.12 Hormone detection

Prolactin (Roche; 07027737190), insulin (INS) (07027559190), cortisol (07027150190), oestradiol (07027249190), free T3 (09005811190), progesterone (07027699190), thyroid stimulating hormone (08443432190) and growth hormone (07027486190) were detected on the Roche cobas e801 automatic electrochemical luminescence analyser using corresponding kits. For specific steps, please refer to the instructions of the respective kit and standard operating procedures.

### 2.13 Cell counting kit‐8 assay and cell viability assay

The proliferation of cells was detected using a cell counting kit (CCK)‐8 (RM02823; ABclonal) assay following the manufacturer's instructions. The cells (1 × 10^3^ cells/well) were seeded in a 96‐well plate with 100 µL of DMEM supplemented with 10% FBS. After incubating for 48 h, the CCK‐8 reagent (10 µL) was added to each well and cultured continuously for 1 h at 5% CO_2_. The absorbance rate at 450 nm was measured using a microplate reader (TECAN; F50). All experiments were repeated three times. Cell viability was determined by the CCK8 assay; liposarcoma and fibrosarcoma cells (5000 cells per well) were seeded in a 96‐well plate and cultured with or without drug‐treated medium for a specific period of time. The CCK8 kit was used according to the manufacturer's instructions.

### 2.14 Colony formation assays

HT1080 or SW872 cells were seeded in six‐well plates and maintained for 2 weeks. The colonies were then fixed with 4% paraformaldehyde (DingGuo, Cat No: AR‐0211; Beijing, China) and stained with .5% crystal violet. The total number of colonies was calculated using image pro plus.6.0.

### 2.15 Immunohistochemistry

Tissue sections were dewaxed in xylene and then hydrated in graded alcohol solutions and distilled water. Immunohistochemical staining was performed using antibodies against SOX4, PRLR and CREB1, according to the manufacturers’ recommendations. Sepia staining was considered as positive staining. A digital tissue biopsy scanner from 3DHISTECH, Hungary, was used to scan the tissue microarray, and Aipathwell, a digital pathological image analysis software from Servicebio, was used to automate the analysis of the collected images mentioned above. The evaluation items included: positive cell ratio = number of positive cells/total number of cells; H‐SCORE = ∑(*pi* × *i*) = (percentage of weak intensity × 1) + (percentage of moderate intensity × 2) + (percentage of strong intensity × 3); IRS = SI (positive intensity) × PP (positive cell ratio). SI was divided into three grades: grade 0 represented no positive staining; grade 1 represented light yellow weak positivity; grade 2 represented brown yellow medium positivity; grade 3 represented tan strong positivity. PP was divided into four levels: level 0 indicated a range between no positivity and up to 5%; level 1 indicated a range between 6% and up to 25%; level 2 indicated a range between 26% and up to 50%; level 3 indicated a range between 51% and up to 75%; level 4 indicated a range greater than 75%.

### 2.16 Western blot analysis

The protein from cells or tissues were lysed using RIPA lysis buffer (P0013B; Beyotime) supplemented with proteinase inhibitor cocktail (TargetMol; Cat3C0001). The protein concentration was measured using a bicinchoninic acid assay. Total proteins (20 µg) were subjected to SDS‐PAGE and transferred onto polyvinylidene difluoride membrane. The membranes were blocked with defatted milk at room temperature for 1 h and then incubated with antibodies overnight at 4°C, including SOX4 (Abcam; Cat#ab70598). Subsequently, the membranes were incubated with HRP‐conjugated secondary antibodies: goat anti‐rabbit IgG antibody (Gen‐Script; Cat#A00098) (dilution 1:5000), goat anti‐mouse IgG antibody (GenScript; Cat#A00160) (dilution 1:5000) and visualised by chemiluminescence.

### 2.17 In vitro white adipocyte differentiation

For white adipocyte differentiation of nonadipogenic lineages, confluent cells were induced by treatment with a cocktail (.5 mM isobutylmethylxanthine, 1 µM dexamethasone, 10 µg/mL INS and .5 µM rosiglitazone) for 2 days and then were treated with maintenance media (10 µg/mL INS) containing high‐glucose DMEM with 10% FBS and 10 mg/mL INS. The maintenance media was changed every day until harvest.

### 2.18 RNA purification, reverse transcription and qPCR

Total RNA was extracted from tissues and cells with TRIzol reagent (Invitrogen) according to the manufacturer's instructions. 1 µg of total RNA was converted to cDNA with 4×Hifair® III SuperMix plus mix using Hifair III 1st Strand cDNA Synthesis SuperMix (Yeasen; 11141ES). Quantitative PCR was performed in the SLAN‐96S (Zeesan) with specific primers and Hifair® III One Step RT‐qPCR SYBR Green Kit (Yeasen; 11143ES) according to the manufacturer's instructions. The relative abundance of mRNAs was standardised with GAPDH as the invariant control. All real‐time qPCRs were carried out in triplicate. qPCR primers are shown in Table S7.

### 2.19 RNA‐seq and analysis

Transcriptome sequencing of patient's serum‐treated cells was conducted by BGI (BGI, Shenzhen, China) according to a published procedure.^16^ RNA counts were imported into R (v4.1.3), and normalisation for library size and regularised logarithmic transformation of counts were performed using DESeq2 (v1.34.0). A volcano plot was drawn using ggplot2 (v3.3.6). The heatmap was drawn using pheatmap (v1.0.12) based on the gene expression in different samples. To gain insight into the phenotypic changes, Gene Ontology (GO) enrichment analysis and KEGG pathway analysis of annotated differentially expressed genes (DEGs) were performed using Phyper based on the hypergeometric test from GO website (http://www.geneontology.org/) and KEGG database website (https://www.kegg.jp/), respectively. The significance levels of terms and pathways were corrected by *Q* value with a rigorous threshold (*Q* value ≤ .05).

### 2.20 Statistical analysis

For targeted metabolomics, lipidomics, proteomics and transcriptomics data, we performed analysis using the Metware Cloud, a free online platform for data analysis (https://cloud.metware.cn); all missing data should be filled with the minimum value. For the screening of differentially expressed metabolites (DEMs) in targeted metabolomics, a two‐sided Student's *t*‐test was employed with Benjamini–Hochberg (BH) false discovery rate (FDR) correction for multiple testing. Metabolites exhibiting a fold change (FC) > 1.5 and a BH‐adjusted *p* value < .05 were identified as DEMs. For the screening of differentially expressed lipids (DELs) in lipidomics, two‐sided Student's *t*‐tests were conducted with BH FDR multiple testing corrections. Lipids exhibiting a FC > 2 and a BH‐adjusted *p* value < .05 were identified as DELs. For the screening of differentially expressed proteins (DEPs), Welch's *t*‐tests were performed with BH FDR multiple testing corrections. Proteins exhibiting a FC > 1.3 and a BH‐adjusted *p* value < .05 were defined as DEPs. For the screening of DEGs in RNA‐seq, two‐sided Student's *t*‐tests were conducted, with BH FDR multiple testing corrections. Genes with a FC > 1.5 and a BH‐adjusted *p* value < .05 were defined as DEGs. Partial least squares discriminant analysis (OPLS‐DA) was used to discern between groups based on their metabolic, RNA‐seq and proteomic profiles. Following data normalisation, an OPLS‐DA model was constructed, which revealed robust separation between control and treatment groups. The model showed excellent predictive capability with *R* ^2^ *Y* and *Q* ^2^ values of *X* and *Y*, respectively. A number of molecules with high VIP scores were identified as potential biomarkers distinguishing these two conditions: Analysis software package and version: R version 3.5.1; function package and version: MetaboAnalystR 1.0.1; basic analysis parameters: log transformation, true; 0 value replacement, true; VIP threshold, 1.0. Volcano plots were drawn to visually display the distribution of differentially expressed molecules in each group. The red, blue and grey dots indicate the up‐regulated molecules, the down‐regulated molecules and the molecules in which the difference in expression was non‐significant, respectively. The volcano plot was performed using the Metware Cloud, a free online platform for data analysis. Analysis software package and version: Python 3.6.6. Function package and version: pandas 0.23.4. Basic analysis parameters: default parameters: FC threshold 2.0, *p* value threshold .05. Heatmap was performed using the Metware Cloud, a free online platform for data analysis: analysis software package and version: R version 4.2.0; function package and version: ComplexHeatmap 2.12.0. The enrichment analysis of metabolites was conducted using the online software MetaboAnalyst 6.0. After importing the metabolites and lipids, the KEGG database was selected for pathway enrichment analysis. The chord diagram analysis of targeted metabolomics and proteomics was conducted using the online software Metware. The Pearson method was selected for the calculation of correlation coefficients, with a correlation coefficient threshold set at.8 and a significance *p* threshold set at.05. The analysis software package and version: R version 3.5.1; function package and version: stats 3.5.1.

All data were collected from more than three independent experiments and expressed as the mean ± standard error of the mean. Statistical significance was determined using *t*‐tests. Graphing was performed using GraphPad Prism 8 software (GraphPad Software, San Diego, CA, USA). The data are presented as means ± SD. A *p* value < .05 was considered statistically significant and is labelled with one asterisk (*), a *p* value < .01 is labelled with two asterisks (**) and a *p* value < .001 is labelled with three asterisks (***).

## 3 RESULTS

### 3.1 Integrated multi‐omics reveals galactose metabolic reprogramming in RDDLPS

**FIGURE 1:**
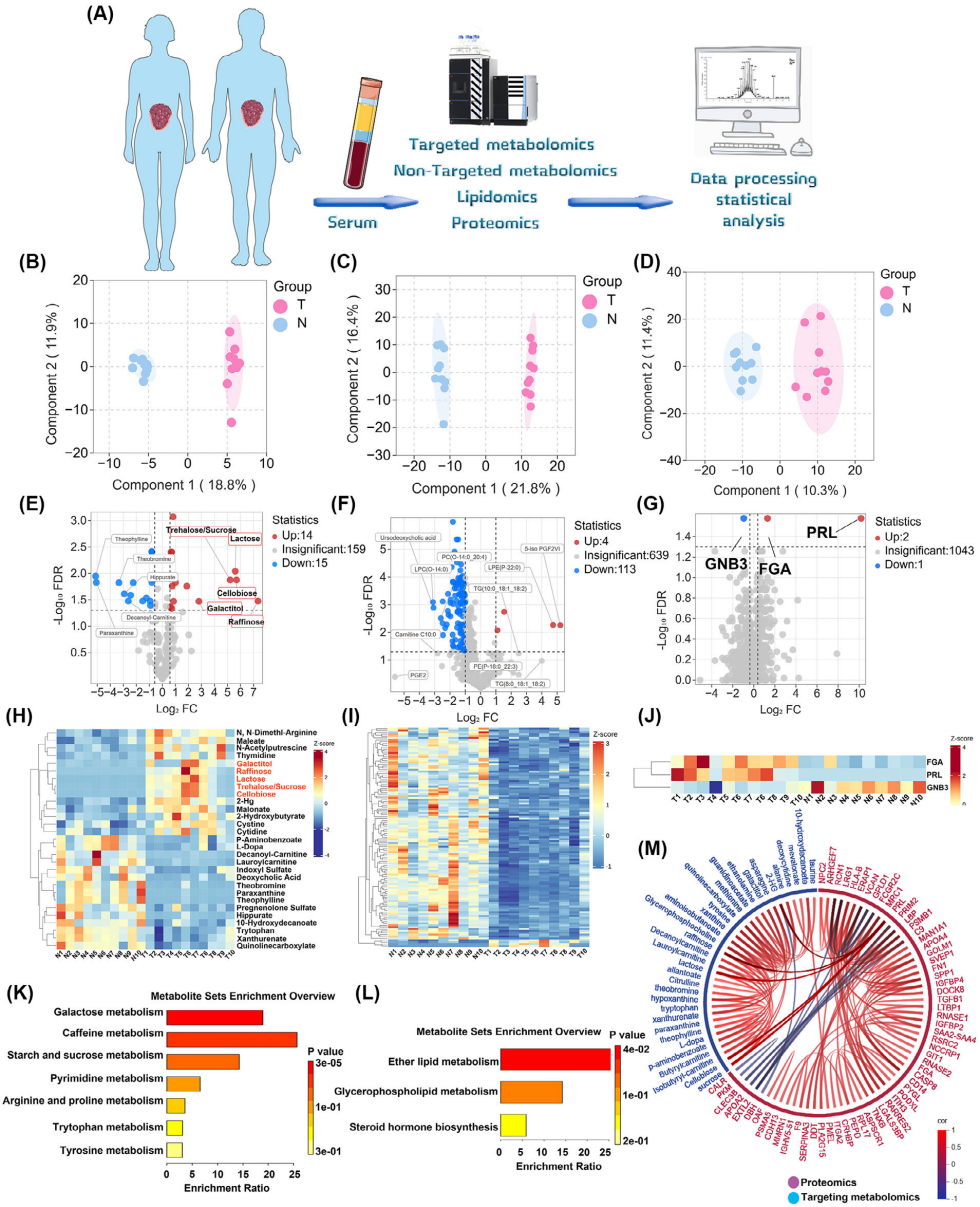
Integrated multi‐omics profiling reveals metabolic dysregulation in retroperitoneal dedifferentiated liposarcoma (RDDLPS). (A) Schematic workflow of clinical specimen acquisition and multi‐dimensional analytical strategy. (B–D) Orthogonal partial least squares‐discriminant analysis (OPLS‐DA) score plots showing distinct clustering between DDLPS patients (*n* = 10) and healthy controls (*n* = 10) in (B) targeted metabolomics (*R* ^2^ *Y*:.991, *Q* ^2^:.828), (C) lipidomics (*R* ^2^ *Y*:.996, *Q* ^2^:.849) and (D) proteomics datasets (*R* ^2^ *Y*:.951, *Q* ^2^:.437). (E–G) Volcano plot of differentially expressed. (E) Metabolites (FC > 1.5, BH‐adjusted *p* value < .05), (F) lipids (FC > 2, BH‐adjusted *p* value < .05) and (G) proteins (FC > 1.3, BH‐adjusted *p* value < .05), red: up; blue: down; grey: insignificant. (H–J) Heatmaps of the differentially expressed molecules: (H) targeted metabolites, (I) lipids, (J) proteins. (K and L) KEGG pathway enrichment bar charts for (K) metabolic pathways and (L) lipid‐related pathways. (M) The chord diagram of proteomics and targeted metabolomics (correlation coefficient threshold,.8; *p* value threshold,.05).

Given the current lack of comprehensive serum multi‐omics studies in RPS, we conducted an integrated multi‐omics analysis – including targeted metabolomics (Table S1), lipidomics (Table S2) and proteomics (Table S3) – on serum samples from patients with RDDLPS, a prevalent RPS subtype (Table S4, clinical cohort details). Ten RDDLPS and 10 healthy control serum samples were randomly selected for analysis (Figure 1A). Following data standardisation, mass spectrometry data underwent log2 transformation to normalise skewed distributions and mitigate heteroscedasticity. OPLS‐DA demonstrated distinct clustering patterns, with components 1 and 2 explaining variability as follows: 11.9%/18.8% in metabolomics, 16.4%/21.8% in lipidomics and 10.8%/10.1% in proteomics (Figure 1B–D). The results revealed 29 DEMs in targeted metabolomics, among which 14 were up‐regulated and 15 down‐regulated. In lipidomics, 117 differential lipids were identified, with four up‐regulated and 113 down‐regulated. Proteomics analysis detected three DEPs, comprising two up‐regulated and one down‐regulated. Volcano plots and heatmaps further characterised these molecular signatures (Figure 1E–J). Notably, RDDLPS patients exhibited elevated serum carbohydrate metabolites (lactose, galactitol, raffinose, cellobiose, sucrose) compared with controls, while caffeine and its derivatives (theobromine, paraxanthine, theophylline) showed significant down‐regulation in targeted metabolomics. Proteomic profiling revealed up‐regulation of PRL, FGA and down‐regulation of GNB3 (Figure 1E–J). Pathway enrichment analysis highlighted significant alterations across galactose metabolism, caffeine metabolism, starch and sucrose metabolism, ether lipid metabolism and glycerophospholipid metabolism (Figure 1K,L). Integrative multi‐omics analysis revealed positive correlations (*r* > .8, *p* < .05) between PRL and carbohydrate metabolites (lactose, galactitol, raffinose, cellobiose, sucrose) (Figure 1M).

### 3.2 Patients with RPS exhibit elevated serum PRL levels

**FIGURE 2:**
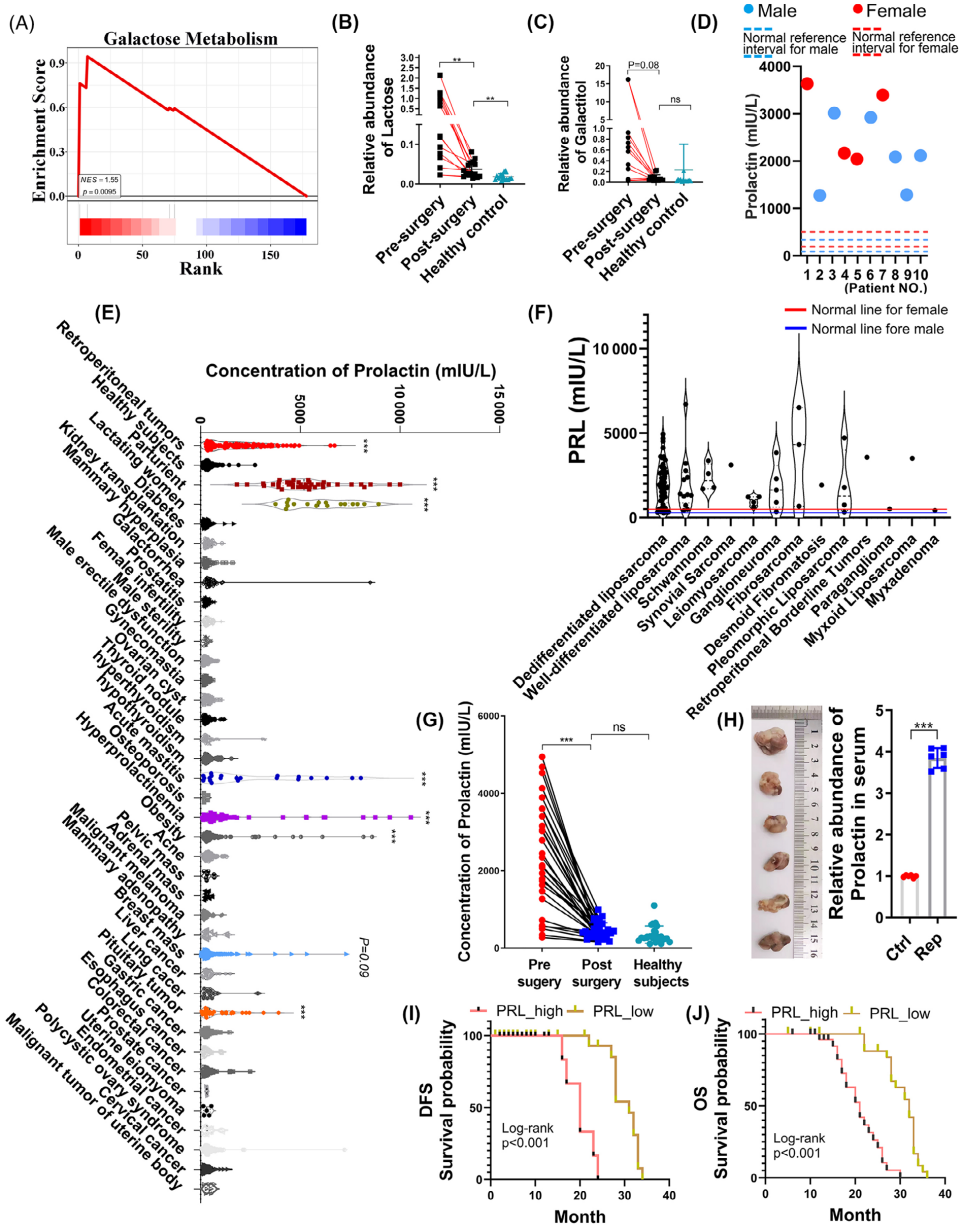
Analysis of PRL in clinical serum specimens. (A) Genome‐wide enrichment analysis (GSEA) demonstrates significant activation of galactose metabolism pathway in RDDLPS serum samples. (B and C) Mass spectrometry quantification of (B) lactose and (C) galactitol levels across clinical cohorts: preoperative (*n* = 15), post‐operative (*n* = 15) and healthy controls (*n* = 15). (D) PRL concentration profiling in multi‐omics discovery cohort, *n* = 10. (E) Retrospective and quantitative analyses of serum PRL levels in patients and healthy control: retroperitoneal tumours (*n* = 100), healthy subjects (*n* = 169), other disease categories and corresponding case counts (Table S4). (F) Quantitative analyses of serum PRL levels in different types of retroperitoneal tumours patients, dedifferentiated liposarcoma (*n* = 57), well‐differentiated liposarcoma (*n* = 17), schwannoma (*n* = 4), synovial sarcoma (*n* = 1), leiomyosarcoma (*n* = 4), ganglioneuroma (*n* = 5), fibrosarcoma (*n* = 3), desmoid fibromatosis (*n* = 1), pleomorphic liposarcoma (*n* = 4), retroperitoneal borderline tumours (*n* = 1), paraganglioma (*n* = 1), myxoid liposarcoma (*n* = 1) and myxadenoma (*n* = 1). (G) Longitudinal assessment of surgical intervention efficacy in patients with RPS: pre‐surgery (*n* = 30) versus post‐surgery (*n* = 30) PRL levels compared with healthy subjects (*n* = 24). (H) Analysis of murine PRL in serum after retroperitoneal inoculation of murine fibrosarcoma cell line (WEHI164) in mice, Ctrl: none sarcoma bearing, Rep: retroperitoneal sarcoma bearing mice (*n* = 7). (I and J) Log‐rank survival curves demonstrate PRL‐associated (I) disease‐free survival (DFS) and (J) overall survival (OS) outcomes in PRL‐high (*n* = 31) and PRL‐low (*n* = 31) groups. Data expressed as mean ± SD unless specified; **p < .01, ***p < .001 by two‐tailed Student's *t*‐test; ns: not significant.

GSEA of metabolic signatures revealed significant up‐regulation of galactose metabolism pathways (Figure 2A). Validation through expanded cohort analysis (including post‐operative RDDLPS serum) demonstrated sustained elevation of these carbohydrates in pre‐surgery samples versus controls and post‐surgery specimens (Figures 2B,C and S1A–C).

Lactose biosynthesis in mammary epithelium involves an evolutionarily conserved pathway regulated by PRL, glucocorticoids, INS, triiodothyronine (T3) and epidermal growth factor (EGF), with PRL serving as the master regulator (Figure S2). Serum hormonal quantification confirmed universal hyperprolactinaemia (exceeding clinical thresholds) in all RDDLPS cases (Figures 2D and S1D–I), aligning with our previous proteomics findings of significantly elevated PRL. Extended analysis of untreated patients from Xiang'an Hospital (Xiamen University, 2019–2023) identified pathological PRL elevation in retroperitoneal tumours, hyperprolactinaemia, acute mastitis and breast neoplasms. Cross‐cohort comparisons (lactation, parturition, common tumours) revealed significant PRL increases during late gestation, lactation and in breast/pituitary malignancies versus normative ranges (Figure 2E). We systematically evaluated 100 patients encompassing diverse histological subtypes‐including dedifferentiated liposarcoma (DDLPS), well‐differentiated liposarcoma (WDLPS), schwannoma, synovial sarcoma, leiomyosarcoma, ganglioneuroma, fibrosarcoma, desmoid fibromatosis, pleomorphic liposarcoma, retroperitoneal borderline tumours, paraganglioma, myxoid liposarcoma and myxadenoma (Figure 2F). In summary, hyperprolactinaemia was clinically confirmed in 90 cases (90%), encompassing both malignant sarcomas and benign tumours. The patient's serum level after the operation decreased significantly compared with that before the operation, and there was no significant difference from that of the normal population (Figure 2G). Control experiments with non‐neoplastic laparotomy patients (appendectomy/cholecystectomy/gastric repair) confirmed surgical trauma does not significantly alter PRL levels (Figure S3). In murine models, orthotopic implantation of fibrosarcoma WEHI164 cells induced significant PRL elevation versus controls (Figure 2H). Clinical survival analysis using median serum PRL as cutoff revealed significantly reduced disease‐free survival (DFS; *p* < .001) and overall survival (OS, *p* < .001) in high‐PRL RDDLPS patients (Figure 2I–J). External validation using published RLPS RNA‐seq data^17^ confirmed worse DFS (*p* = .019) and OS (*p* = .023) in PRL‐high subgroups (Figure S4). These findings position hyperprolactinaemia as a common occurrence among patients with various retroperitoneal tumours, with PRL levels decreasing post‐operatively. Additionally, patients with retroperitoneal liposarcoma and high PRL levels have a poorer prognosis.

### 3.3 Sarcoma cells demonstrate the capacity for PRL production and release

**FIGURE 3:**
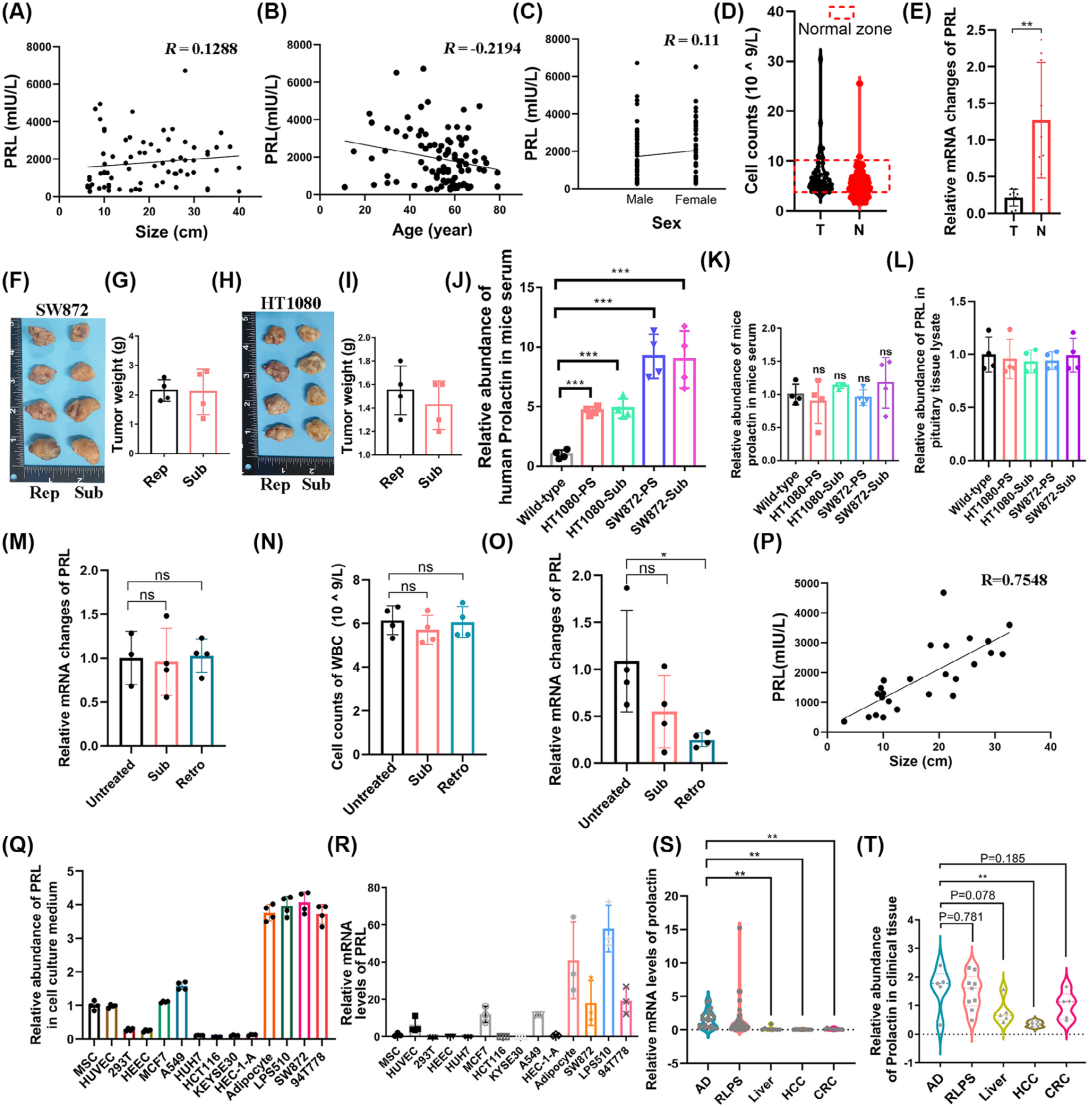
Investigation of PRL origin. (A–C) Spearman correlation analysis between serum PRL levels and (A) tumour size (*n* = 73, *r* = −.12), (B) patient age (*n* = 99, *r* = −.219) and (C) gender distribution (*n* = 100,.11) in retroperitoneal tumour cohort. (D and E) Comparative analysis of (D) leukocyte counts (*n* = 98) and (E) PBMC PRL mRNA expression (*n* = 9) between tumour patients with retroperitoneal tumours (T) and healthy controls (N). (F–J) Measurement of human sarcoma cells derived PRL in tumour xenograft mouse serum: tumour weight of orthotopic and subcutaneous xenograft models using liposarcoma cells (F and G) and fibrosarcoma cells (H and I), human‐derived PRL in mice serum (J), murine‐derived PRL in mice serum (K), *n* = 4, Rep: retroperitoneal orthotopic xenograft, Sub: subcutaneous xenograft. (K) Analysis of the relative expression level of PRL in the serum in the tumour‐bearing mouse model, *n* = 4. (L and M) Analysis of the relative expression level of PRL mRNA and protein in the pituitary gland in the tumour‐bearing mouse model, *n* = 4. (N and O) Peripheral leukocyte profiling showing (N) cell counts and (O) intracellular PRL mRNA in tumour‐bearing mice, *n* = 4. (P) Spearman correlation analysis between serum PRL levels and tumour size, the sarcoma tissue used for analysis is similar to the screened tissue in terms of cellular morphology and tissue structure (*n* = 25, *r* = .7548). (Q and R) In vitro assessment of (Q) secreted PRL and (R) cellular PRL mRNA in human sarcoma cell lines, *n* = 3. (S and T) Analysis of PRL protein and mRNA levels in clinical tissues, AD: adipocyte (*n* = 23), RLPS: retroperitoneal liposarcoma (*n* = 23), HCC: hepatocellular carcinoma (*n* = 23), CRC: colorectal cancer (*n* = 23). Data expressed as mean ± SD unless specified; *p < .05, **p < .01, ***p < .001 by two‐tailed Student's *t*‐test; ns: not significant.

Building upon these findings, we systematically investigated potential correlations between PRL elevation and clinical parameters including tumour size, age and gender. Correlation analysis revealed no significant associations between serum PRL levels and these variables (Figure 3A–C). To elucidate the origin of hyperprolactinaemia in RLPS, we evaluated three potential sources: leukocytic production, pituitary secretion and autonomous tumour synthesis. Initial assessment of leukocytic contributions showed 69.3% of patients maintained white blood cell counts within normal ranges (Figure 3D), while qPCR analysis demonstrated significantly reduced PRL mRNA expression in patient leukocytes compared with healthy controls (Figure 3E). To assess pituitary involvement, orthotopic retroperitoneal and subcutaneous xenograft models were established using human liposarcoma (SW872) and fibrosarcoma (HT1080) cell lines. When tumours reached ∼1 cm diameter (Figure 3F–I), tumour‐bearing mice exhibited significantly elevated human‐derived serum PRL compared with non‐tumour controls, with liposarcoma xenografts showing greater PRL elevation than fibrosarcoma models (Figure 3J). Notably, murine‐derived PRL remained unchanged in serum across groups (Figure 3K), and no tumour‐related alterations in pituitary PRL mRNA and protein expression were observed (Figure 3L,M). Leukocyte counts were comparable between groups (Figure 3N); tumour‐bearing mice exhibited reduced leukocytic PRL mRNA expression (Figure 3O).

It is noteworthy that RPSs exhibit substantial heterogeneity. Taking retroperitoneal liposarcoma as an example, we performed HE staining on a retroperitoneal liposarcoma tissue microarray. The staining results revealed significant heterogeneity in both cellular morphology and tissue architecture. Tumour size showed no correlation with the number of tumour cells within the tumour tissue (Figure S5). Although Figure 3A indicates no significant correlation between tumour size and prolactin levels, this does not imply that the quantity of RPS cells is unrelated to serum prolactin levels in patients. It merely suggests that retroperitoneal tumours do not regulate serum prolactin levels through physical volume. To further analyse the correlation between cellularity‐based sarcoma size and serum prolactin, we selected retroperitoneal liposarcoma tissues with similar cellular and histological features and conducted a correlation analysis between the corresponding patients’ prolactin levels and tumour dimensions (Figure S6). The results demonstrated a positive correlation between serum prolactin levels and tumour size in this subset of patients (Figure 3P; *R* = .7548). Based on these findings, we hypothesise that the abnormally elevated serum prolactin in patients may originate from the sarcoma tissue. Subsequently, comprehensive screening of PRL secretion across diverse cell types revealed adipocytes and liposarcoma cells (LPS510, SW872, 94T778) secreted significantly higher PRL levels compared with MSCs, epithelial lineages and other cancer cells via ELISA (Figure 3Q). Additionally, we successfully isolated liposarcoma cells (designated as XMU‐RC‐1) from clinically resected dedifferentiated liposarcoma tissue and confirmed that XMU‐RC‐1 similarly secretes PRL (Figure S7). Corresponding qPCR analysis confirmed enhanced PRL mRNA expression in these adipocytic and liposarcoma lineages (Figure 3R). Clinical validation using human tissues demonstrated markedly elevated PRL RNA and protein levels in adipose and liposarcoma tissues versus hepatic, colorectal and adjacent normal tissues (Figure 3S,T). These findings indicate that liposarcoma tissues represent novel extrapituitary sources of PRL.

### 3.4 SOX4 drives PRL gene transcription via direct promoter activation

**FIGURE 4:**
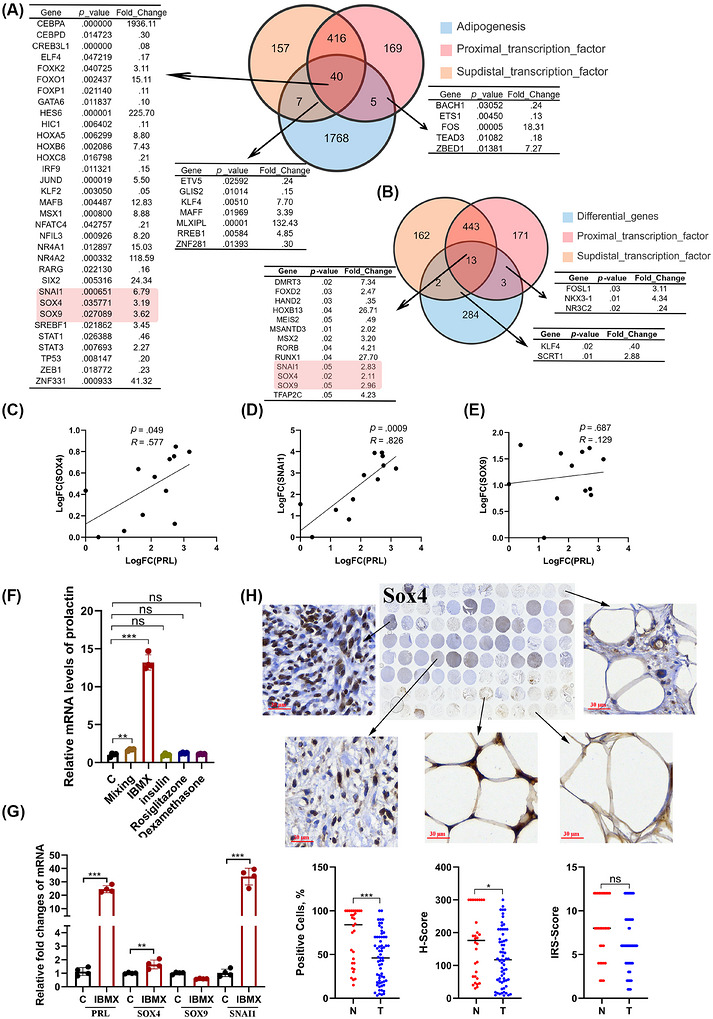
Systematic identification of PRL‐regulating transcriptional machinery in liposarcoma. (A and B) Intersection analysis of candidate transcription factors (TFs): (A) Venn diagram integrating TFs from mesenchymal stem cell (MSC) versus adipocyte differential genes, transcriptors in distal transcriptional regions of PRL gene, and transcriptors in proximal transcriptional regions of the PRL gene (*p* > .05, fold change > 2). (B) Comparative intersection of TFs from PRL‐high versus PRL‐low (cutoff criteria: median) sarcomas, transcriptors in distal transcriptional regions of PRL gene and transcriptors in proximal transcriptional regions of the PRL gene. (C–E) Correlation analysis between PRL mRNA and (C) SOX4 (*n* = 12), (D) SOX9 (*n* = 12), (E) SNAI1 (*n* = 12) in clinical specimens, Log FC = Log10 fold change (sample X to sample minimum), fold change sample X to sample minimum = power (2, −[Ct(X) − Ct(gapdh)] − [Ct(X) − Ct(gapdh)]max). (F) Pharmacological induction assay: PRL mRNA fold‐change in SW872 cells treated with adipogenic cocktails (IBMX.5 mM, insulin 5 µg/mL, rosiglitazone 2 µM, dexamethasone 1 µM) versus DMSO control (*n* = 4). (G) qPCR analysis of PRL and candidate TFs under IBMX (.5 mM) treatment (*n* = 4). (H) Tissue microarray validation: SOX4 protein expression quantification by immunohistochemical analysis in adipose tissue (*n* = 30), RWDLPS (*n* = 20) and RDDLPS (*n* = 50). Data expressed as mean ± SD unless specified; **p < .01, ***p < .001 by two‐tailed Student's *t*‐test; ns: not significant.

Given the shared mesenchymal origin of retroperitoneal liposarcoma and adipocytes, combined with their demonstrated PRL hypersecretion, we hypothesised that transcriptional regulators governing PRL expression are activated during both adipogenic differentiation and tumourigenesis of sarcoma. Differentiation experiments corroborated this adipocyte‐associated secretory capacity, with human MSCs and 3T3‐L1 murine preadipocytes showing significant PRL secretion elevation upon adipogenic induction (Figure S8A,B). To identify candidate regulators, we implemented a multi‐omics strategy: transcriptomic analysis of MSC‐derived beige adipocyte differentiation (GSE125331 dataset); PRL‐stratified sarcoma RNA‐seq profiling (GSE71119 dataset); JASPAR database predictions of PRL regulatory region‐binding TFs. The Venn diagram summarises the potential transcription factors of PRL that are differentially expressed during the adipogenic differentiation of MSC cells (Figure 4A), as well as the differential PRL‐associated transcription factors identified between the high‐ and low‐PRL expression groups in sarcoma tissue (Figure 4B). We found that the three transcription factors, SOX4, SOX9 and snai1, simultaneously emerged in the screening process of the two sets of differentially expressed transcription factors mentioned above.

Subsequently, we randomly extracted total RNA from 12 cases of dedifferentiated liposarcoma tissues. The expression levels of PRL, SOX4, SOX9, SNAI1 and GAPDH were quantified via qPCR. The ΔCt values were calculated by subtracting the Ct value of GAPDH from the Ct values of PRL, SOX4 and SOX9, respectively. The sample with the highest ΔCt value was designated as the reference. The ΔΔCt values were then derived by subtracting the reference ΔCt from the ΔCt of each sample. Finally, relative expression levels were determined and subjected to logarithmic transformation for subsequent correlation analysis. Tissue validation demonstrated positive PRL expression correlations with SOX4 and SNAI1, but not SOX9 (Figure 4C–E). Adipogenic induction experiments showed IBMX‐mediated co‐upregulation of PRL, SOX4 and SNAI1 in MSCs (Figure 4F,G), while SNAI1 exhibited minimal baseline expression (Ct > 40). A Ct value exceeding 40 in the qPCR process typically indicates minimal gene expression or susceptibility to non‐specific amplification. Consequently, SOX4 has been designated as the primary subject for subsequent investigation. Clinical assessments confirmed elevated SOX4 levels in both liposarcoma and adjacent adipose tissue (Figure 4H); these results enhance the feasibility of our research focus.

**FIGURE 5:**
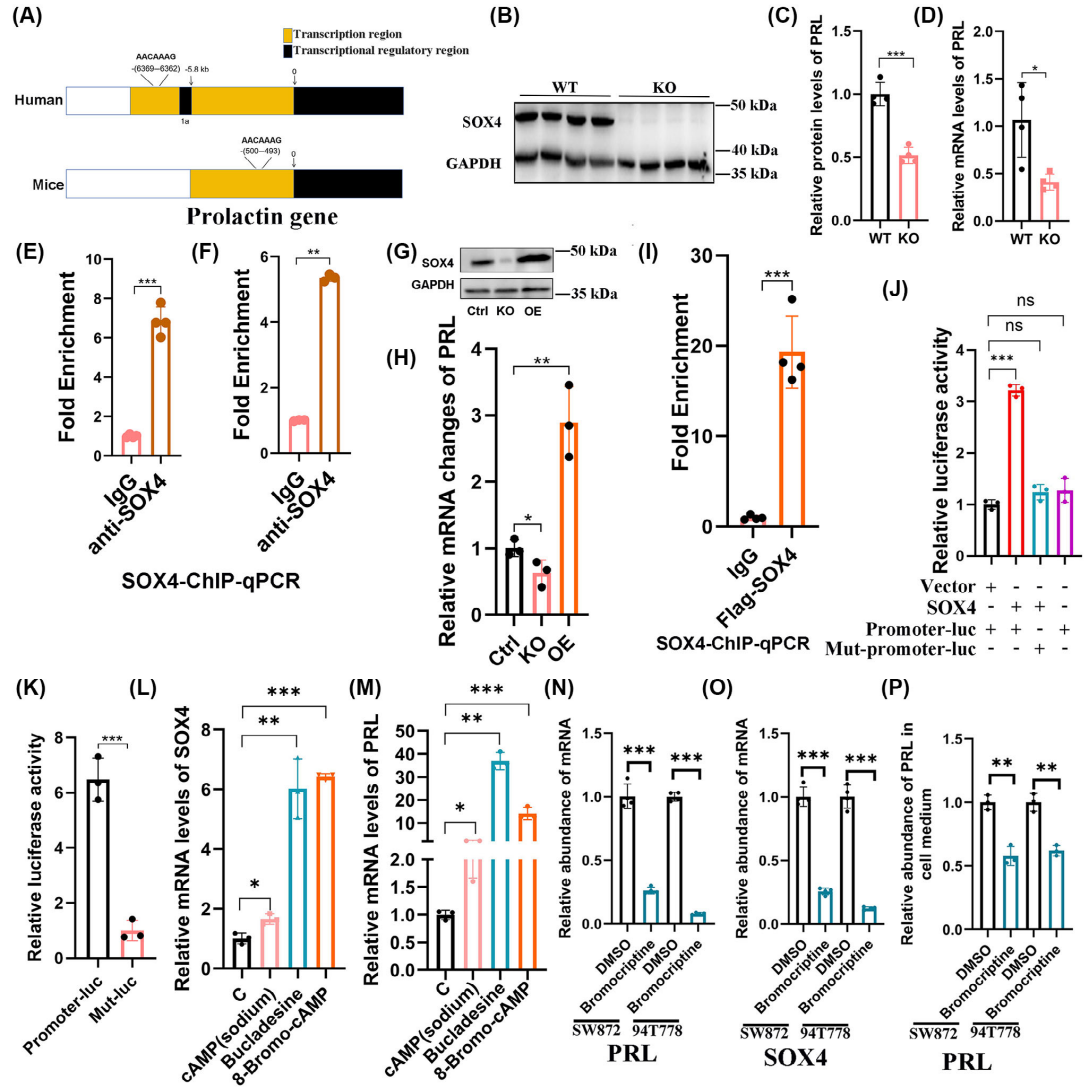
Mechanistic elucidation of SOX4‐mediated transcriptional activation of PRL. (A) Genomic architecture of PRL regulatory elements, highlighting the distal exon 1a of PRL and the conserved SOX4 binding sequence AACAAAG. (B) Adipocyte‐specific SOX4 knockout efficiency validation in Adipo‐Cre; Sox4 knockout mice versus wild‐type littermates (*n* = 4). (C and D) Concomitant reduction of (C) PRL protein (ELISA) and (D) mRNA (qPCR) in SOX4‐deficient adipose tissue (*n* = 4). (E and F) ChIP analysis demonstrating SOX4 protein occupancy at −493 bp upstream of the PRL promoter in beige adipocytes (E) and 3T3‐L1 cells (F), *n* = 4. (G) Examination of the effects of SOX4 knockout and overexpression on SW872 cells. (H) Changes in PRL mRNA levels in SW872 cells after SOX4 knockout and overexpression, *n* = 3. (I) ChIP analysis revealing SOX4 protein occupancy at −6362 bp upstream of the PRL promoter in SW872 cells, *n* = 4. (J) Relative transcriptional activity of the PRL promoter and SOX4 binding site mutant promoters in 293T cells transfected with vector, SOX4, promoter–luc or Mut–promoter–luc constructs, *n* = 4. (K) Relative transcriptional activity of the PRL promoter and SOX4 binding site mutant promoters in SW872 cells transfected with vector or SOX4 constructs, *n* = 3. (L and M) Analysis of the transcriptional regulation of PRL by SOX4 in response to cAMP, and its analogs bucladesine and 8‐Bromo‐cAmp, *n* = 3. (N and O) qPCR was employed to assess the mRNA expression levels of PRL and SOX4 in bromocriptine‐treated SW872 and 94T778 cells, *n* = 3. (P) The relative concentration of PRL protein in the culture supernatant of bromocriptine‐treated SW872 cells was measured, *n* = 3. Data expressed as mean ± SD unless specified; *p < .05, **p < .01, ***p < .001 by two‐tailed Student's *t*‐test; ns: not significant.

Comparative genomic analysis identified evolutionarily conserved SOX4 binding motifs located at −6362 bp (human) and −493 bp (mouse) relative to the PRL transcriptional start site (Figure 5A). Adipose‐specific SOX4 knockout significantly reduced both PRL protein and mRNA levels (Figure 5B–D). ChIP‐qPCR confirmed SOX4 binding to PRL promoters in human adipose tissue and 3T3‐L1 adipocytes (Figure 5E,F). Genetic manipulation in SW872 liposarcoma cells demonstrated dose‐dependent SOX4 regulation of PRL transcription (Figure 5G,H), with direct promoter binding validated by ChIP‐qPCR (Figure 5I). Luciferase reporter assays functionally validated SOX4‐mediated transcriptional activation through the conserved distal element. Wild‐type PRL promoter constructs exhibited higher luciferase activity than mutant controls in 293T cells (Figure 5J). In SW872 cells, wild‐type constructs showed greater activity versus mutants (Figure 5K). These data establish SOX4 as the master transcriptional regulator coordinating PRL expression during adipogenesis and tumourigenesis of sarcoma.

Given reported cAMP‐mediated PRL regulation via PKA‐dependent/independent pathways in human eosinophils,^18^ therefore, we hypothesise that cAMP agonists can up‐regulate prolactin secretion in liposarcoma cells while concomitantly enhancing the expression of their transcription factors. Pharmacological elevation of intracellular cAMP levels in SW872 cells (using cAMP and its analogs bucladesine and 8‐Bromo‐cAMP) increased PRL secretion and enhanced SOX4 transcriptional activity (Figure 5L,M). Subsequently, we selected bucladesine to further investigate the effects of different concentrations on PRL regulation in SW872 cells. It was found that 5 µM bucladesine significantly up‐regulated PRL secretion (Figure S9A). On this basis, we examined common transcription factors of PRL following bucladesine treatment. The results demonstrated significant up‐regulation of cebp2, stat5a, esr1, esr2 and ets1 after bucladesine administration (Figure S10). Furthermore, we hypothesised that PRL might also be regulated by the dopamine–dopamine receptor pathway. Thus, we also assessed dopamine receptors DRD2 and DRD3 and observed that both were markedly up‐regulated upon bucladesine treatment (Figure S10). Consequently, we explored the regulatory effect of different concentrations of the dopamine receptor agonist bromocriptine on prolactin secretion. The results indicated that 20 µM bromocriptine significantly enhanced PRL secretion in SW872 cells (Figure S9B). As a key protein within the cAMP signalling pathway, CREB1 mRNA levels in SW872 cells remained unaltered despite stimulation with a cAMP agonist (Figure S11). Tissue‐level assessments showed high CREB1 expression in liposarcoma but not adipose tissues (Figure S12). Collectively, these results define a cAMP–SOX4–PRL regulatory axis linking metabolic signalling to transcriptional control in liposarcoma pathogenesis. The conserved SOX4 binding mechanism and cAMP responsiveness provide a molecular bridge between adipogenic differentiation programs and tumour‐associated hyperprolactinaemia.

### 3.5 PRL promotes malignant proliferation and chemoresistance in fibrosarcoma and liposarcoma cell models

**FIGURE 6:**
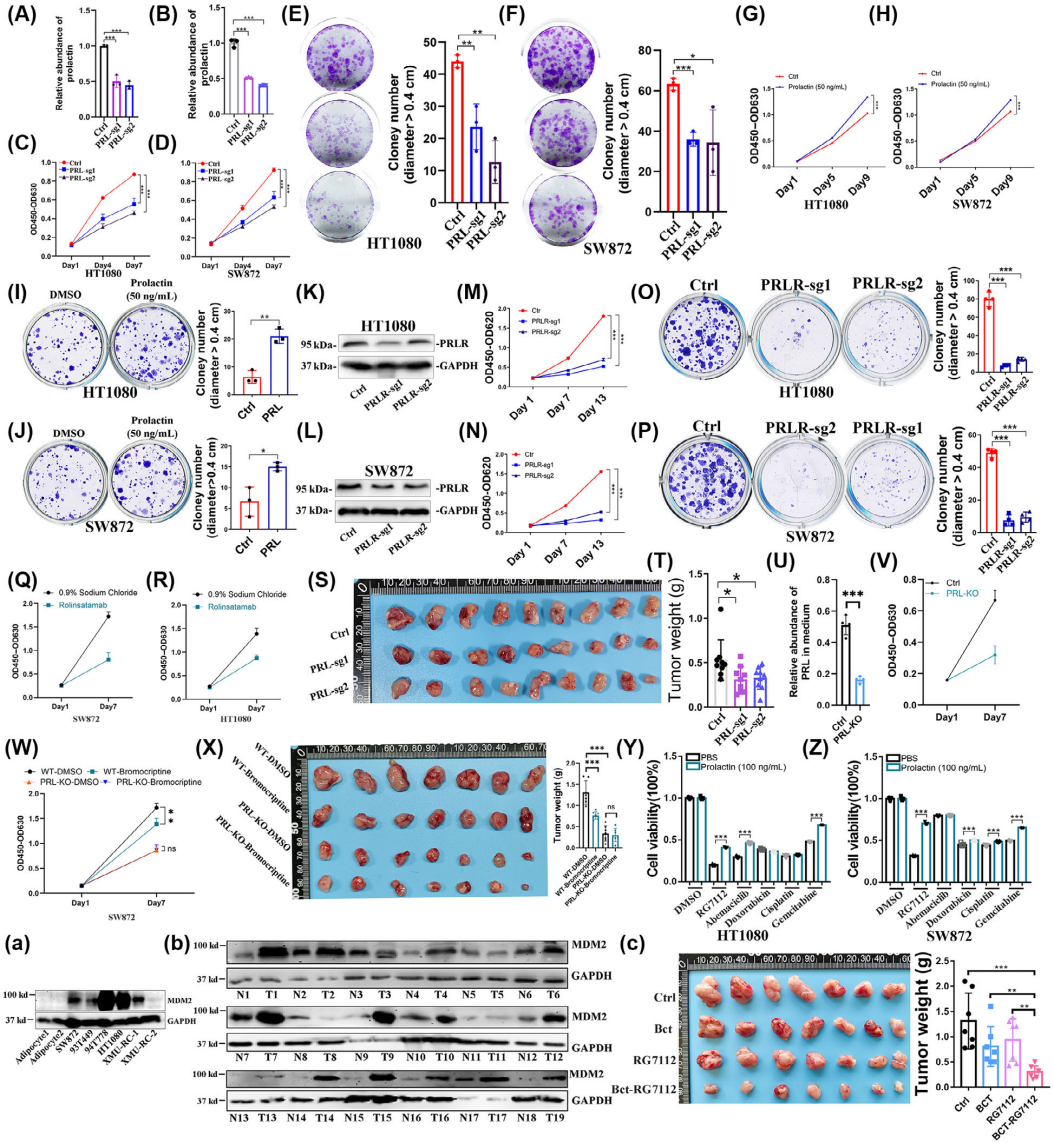
Functional characterisation of PRL‐mediated proliferation and chemoresistance in sarcoma models. (A and B) Secretory PRL quantification by ELISA confirming knockdown efficiency in the culture medium, *n* = 3. (C–F) Growth suppression following PRL depletion: CCK‐8 time‐course assay (*n* = 5) and colony formation capacity (*n* = 3) in PRL‐knockdown models. (G–J) Recombinant PRL (50 ng/mL)‐induced proliferative enhancement: (G and H) CCK‐8 (*n* = 5) and (I and J) colony formation (*n* = 3) in HT1080 and SW872 lines. (K–P) PRLR‐dependent proliferation modulation: (K–N) CCK‐8 dose‐response (*n* = 5) and (O‐P) colony formation (*n* = 3) analysis post‐PRLR perturbation. (Q and R) After treating SW872 and HT1080 cells with PRLR antibody rolinsatamab talirine (20 µg/mL), the effect on cell proliferation was detected by the CCK8 method, with *n* = 5. (S and T) Xenograft tumourigenesis assay demonstrating impaired SW872 growth with PRL knockdown (*n* = 9). (U) A single SW872 clone exhibiting the lowest PRL expression among the pooled PRL‐knockout cells was isolated by limiting dilution cloning, expanded in culture and validated for PRL protein levels via ELISA. (V) Cell proliferation was assessed using the CCK‐8 assay following stable PRL knockout (*n* = 5 biological replicates). (W) Bromocriptine‐mediated antiproliferative effects were evaluated in parallel in wild‐type and PRL‐knockout SW872 cell lines using the CCK‐8 assay (*n* = 5). (X) In vivo efficacy was determined in a subcutaneous xenograft mouse model, wherein tumour growth derived from wild‐type or PRL‐knockout SW872 cells was monitored following bromocriptine treatment, *n* = 7. (Y and Z) Chemosensitisation effects: PRL pretreatment (50 ng/mL) enhances cytotoxicity of RG7112/abemaciclib/doxorubicin/gemcitabine, *n* = 5, RG7112 (10 µM), abemaciclib (5 µM), doxorubicin (2 µM), gemcitabine (10 µM). (a) Western blot analysis was performed to detect MDM2 expression in human adipocytes, liposarcoma cell lines (SW872, 93T449, 94T778), fibrosarcoma cell line HT1080 and clinically isolated liposarcoma cell lines established in our laboratory. (b) Western blot analysis was performed to detect MDM2 in 12 clinical retroperitoneal liposarcoma tissues and its corresponding paracancerous tissues, 6 clinical retroperitoneal fibrosarcoma tissues and corresponding paracancerous tissues. (c) Therapeutic synergy evaluation: bromocriptine combined with RG7112 in WEHI164 fibrosarcoma murine model, RG7112: 100 mg/kg per day, bromocriptine: 10 mg/kg, twice daily, (*n* = 6). Data expressed as mean ± SD unless specified; *p < .05, **p < .01, ***p < .001 by two‐tailed Student's *t*‐test; ns: not significant.

Given the marked PRL elevation observed in retroperitoneal liposarcoma and fibrosarcoma patients, we investigated its functional role in tumour proliferation and chemoresistance using representative HT1080 (fibrosarcoma cell) and SW872 (liposarcoma cell) cell lines. Employing the CRISPR/Cas9 system for PRL gene editing, puromycin selection yielded a drug‐resistant non‐clonal cell pool. Subsequent ELISA analysis of the culture medium revealed a significant down‐regulation of PRL (Figure 6A,B). PRL down‐regulation significantly attenuated both cellular proliferation and colony formation capacity (Figure 6C–F). Conversely, recombinant PRL treatment enhanced proliferation and increased colony formation compared with PBS controls (Figure 6G–J). Assessment of PRLR expression at the tissue level revealed high PRLR levels in both liposarcoma tissues and adjacent adipose tissues (Figure S13). Employing the CRISPR/Cas9 system for PRLR gene editing, puromycin selection yielded a drug‐resistant non‐clonal cell pool. Western blot quantification revealed that in HT1080 cells, sgRNA1 demonstrated a 48% knockdown efficiency, while sgRNA2 exhibited a 23% knockdown efficiency. In SW872 cells, sgRNA1 achieved a 68% knockdown efficiency, and sgRNA2 showed a 66% knockdown efficiency (Figure 6K,L). Genetic suppression of PRLR expression substantially impaired cellular proliferative capacity (Figure 6M–P). In addition, we also found that the antibody drug rolinsatamab targeting PRLR could significantly inhibit the proliferation ability of cells (Figure 6Q,R). In vivo validation using PRL‐down‐regulated SW872 cells demonstrated a reduction in tumour volume compared with controls (Figure 6S), confirming PRL's pro‐proliferative role in malignant progression.

Building upon these findings, we employed monoclonal cloning to isolate SW872 and 94T778 cell sublines exhibiting enhanced PRL knockout efficiency (Figures 6U and S13B). Given that bromocriptine is a clinically approved first‐line dopamine agonist for hyperprolactinaemia – and given our observation that it significantly suppresses prolactin secretion in SW872 cells (Figure S9B) – we next evaluated its antiproliferative effects in both SW872 and 94T778 liposarcoma cells. Bromocriptine treatment markedly inhibited cellular proliferation in parental (i.e., PRL‐intact) SW872 and 94T778 cells (Figure S14A). In contrast, this inhibitory effect was substantially attenuated in PRL‐knockout derivatives (Figures 6V,W and S14A–G), indicating PRL dependence of the response. Consistent with the in vitro results, bromocriptine significantly suppressed tumour growth in xenograft models derived from parental cells but showed diminished efficacy in tumours formed by PRL‐knockout cells (Figure 6X).

To assess physiological relevance, we evaluated patient‐derived sera (5% v/v) in proliferation assays. Preoperative RPS serum significantly enhanced HT1080 and SW872 cell growth versus post‐operative samples in seven out of eight cases (Figures S15 and S16). Similarly, serum from parturients and hyperprolactinemic patients showed greater proliferative capacity than healthy controls (Figure S17A–D). PRLR knockdown abolished these proliferative effects (Figure S17E,F), establishing PRL as a critical mediator of serum‐induced proliferation.

Given RPS's characteristic chemoresistance, we investigated PRL's role in therapeutic responses. We selected the following agents for cytotoxicity testing: cisplatin, a commonly used chemotherapeutic drug; doxorubicin and gemcitabine, standard clinical treatments for RPS; as well as the emerging targeted agents abemaciclib (a CDK4 inhibitor) and RG7112 (an MDM2 inhibitor). The result shows that PRL supplementation reduced cytotoxicity of gemcitabine and MDM2 inhibitors in HT1080 and SW872 cells (Figure 6Y,Z). Preoperative serum similarly attenuated RG7112 efficacy compared with post‐operative samples (Figure S18). Prolactin significantly attenuated cell death induced by multiple pharmacologically distinct MDM2 inhibitors – including Nutlin‐3a, SAR405838 and SP141 – demonstrating a consistent prosurvival role across compound classes (Figure S19A,B). However, in PRLR‐knockout cells, PRL failed to exert any rescue effect (Figure S19C,D). MDM2, a key negative regulator of the tumour suppressor p53, has emerged as a compelling therapeutic target in human cancer. Pan‐cancer analyses reveal that MDM2 expression is significantly elevated in tumour tissues compared with adjacent non‐cancerous tissues across multiple cancer types.^19^ Notably, sarcomas characterised by MDM2 genomic amplification include well‐differentiated liposarcoma/atypical lipomatous tumour, dedifferentiated liposarcoma, intimal sarcoma and low‐grade osteosarcoma.^20^

We initially assessed the expression of MDM2 in human adipocytes (adipocyte1, adipocyte2), liposarcoma cell lines (SW872, 93T449, 94T778, HT1080, XMU‐RC‐1, XMU‐RC‐2), a fibrosarcoma cell line (HT1080) and other type tumour cell lines (colorectal cancer: HCT116; lung cancer: A549; breast cancer: MB‐MDA‐231; cholangiocarcinoma: QBC939; oesophageal cancer: KYSE30) via Western blot analysis. The results revealed that MDM2 was highly expressed in SW872, 93T449, 94T778, HT1080 and XMU‐RC‐1 compared with adipocytes (Figure 6a). High levels of MDM2 expression were also detected in HCT116, A549, MB‐MDA‐231, QBC939 and KYSE30 (Figure S20). We randomly selected 12 cases of retroperitoneal liposarcoma (T1–T12) and six cases of fibrosarcoma (T13–T18), along with adjacent normal adipose tissues, to evaluate MDM2 expression. Compared with the adjacent normal adipose tissues, eight out of 12 liposarcoma tissues and six out of six fibrosarcoma tissues exhibited significantly elevated MDM2 expression (Figure 6b). These findings further underscore the considerable potential of targeting MDM2 in the treatment of RPSs. However, multiple early‐phase clinical trials have failed to show a benefit with MDM2 pathway inhibition for DDLPS.^21^ This suggests the potential involvement of PRL‐mediated MDM2‐associated drug resistance in anti‐MDM2 therapeutic strategies.

Furthermore, following treatment with the MDM2‐p53 targeted inhibitors Nutlin‐3a and RG7112, MDM2 was significantly up‐regulated in SW872, HT1080 and HCT116 cells. In contrast, treatment with SP141, an inhibitor that promotes MDM2 self‐ubiquitination and degradation, led to a marked reduction in MDM2 levels. Interestingly, Nutlin‐3a and RG7112 treatment resulted in up‐regulation of p53 in HT1080 and HCT116 cells, whereas no notable change was observed in SW872 cells (Figure S21A–C). Similarly, MDM2 knockdown induced substantial p53 up‐regulation in HCT116 cells, but not in SW872 cells (Figure S21D,E). These results imply that PRL‐mediated MDM2‐associated drug resistance may operate independently of p53.

Clinically, hyperprolactinaemia is usually treated with bromocriptine mesylate (BCT), which is a dopamine D2/D3 receptor agonist and can effectively inhibit PRL secretion by the pituitary gland. Therefore, we first orally administered bromocriptine to mice and then detected serum PRL levels. We found that BCT could delay tumour formation and prolong survival in the orthotopic model (Figure S22). Additionally, the combination treatment of BCT could enhance the anti‐tumour efficacy of RG7112 (Figure 6c). Notably, this therapeutic strategy could maintain normal body weight, haematological parameters and liver and kidney functions (Figure S23 and Table S5). These findings establish hyperprolactinaemia as a dual driver of liposarcoma/fibrosarcoma proliferation and MDM2‐targeted therapy resistance, while identifying PRL signalling as a therapeutically actionable pathway in RPS management.

### 3.6 PRL/PRLR axis activation drives c‐MYC up‐regulation

**FIGURE 7:**
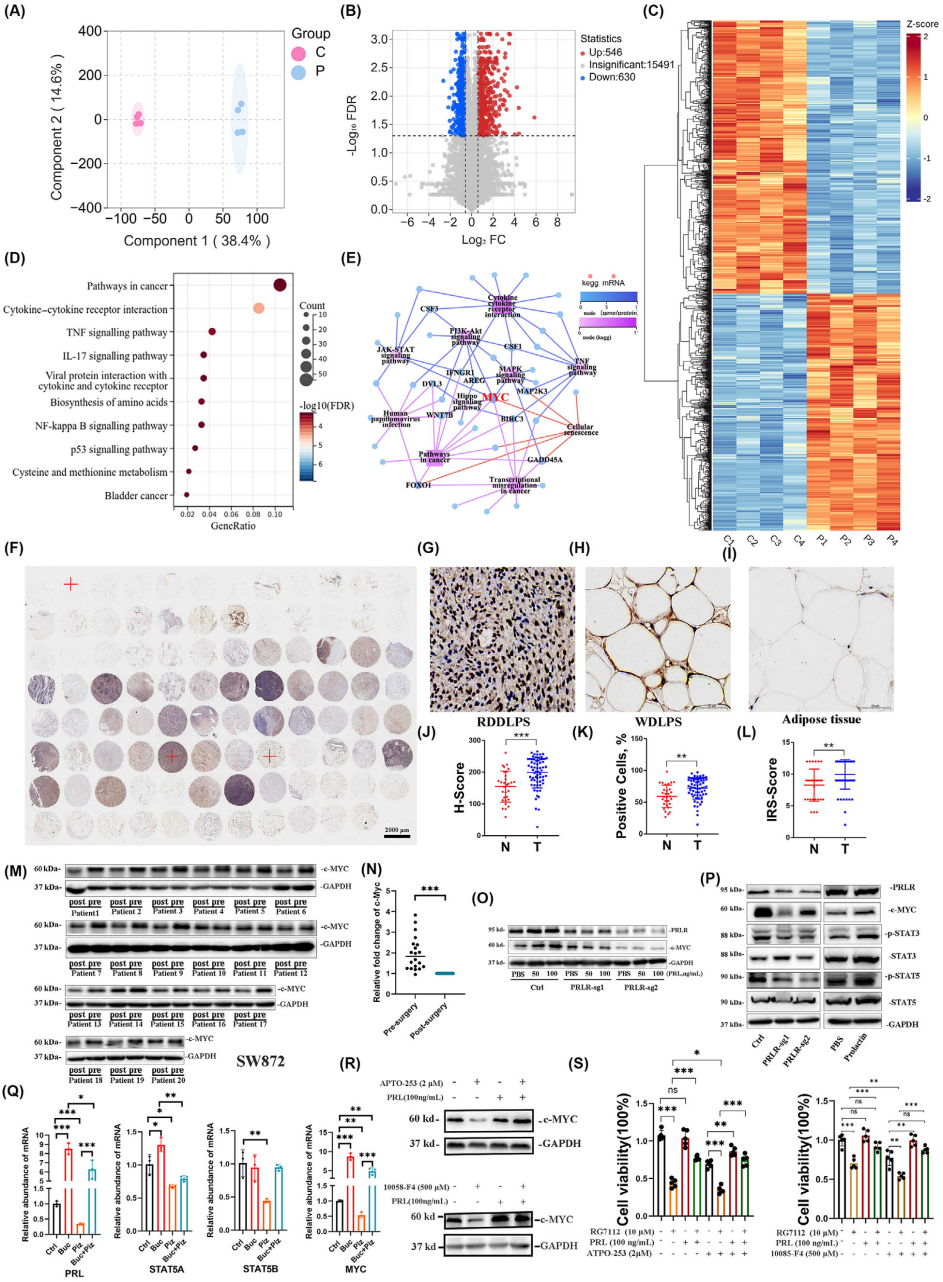
Analysis of the PRL‐regulated pathway. (A) OPLS‐DA score plot distinguishing PRL‐activated (12 h) versus control transcriptional profiles, C: control, P: prolactin, *n* = 4. (B) Volcano plot analysis. Red denotes up‐regulated genes, blue denotes down‐regulated genes and grey denotes insignificant changed genes. (C) Heatmap: a red colour indicates a higher expression level, while blue colour indicates a lower expression level. (D) KEGG pathway enrichment. (E) Regulatory network mapping PRL‐associated genes (circles) to tumour‐related pathways (squares). (F–L) Tissue microarray validation: IHC staining was performed on RLPS tissue and adipose tissue microarray using c‐MYC antibodies. Positive cell%, histochemistry SCORE and IRS are calculated. Adipose tissue: *n* = 30; retroperitoneum well‐differentiated liposarcoma (RWDLPS): *n* = 20; retroperitoneum dedifferentiated liposarcoma (RDDLPS): *n* = 50. T: sarcoma tissue, N: adipose tissue. (M and N) Clinical correlation: pre‐operative serum up‐regulates c‐MYC versus post‐operative serum in SW872. (O) Rescue experiment: PRL‐induced c‐MYC up‐regulation blocked by PRLR knockdown. (P) Western blot analysis of key proteins in the JAK–STAT signalling pathway were detected following down‐regulation of PRLR and addition of recombinant PRL (50 ng/mL) in SW872 cells. (Q) qPCR was performed to assess mRNA expression levels of PRL, STAT5A, STAT5B and MYC in SW872 cells following treatment with the cAMP agonist bucladesine or the STAT5 inhibitor pimozide, alone or in combination. Bucladesine treatment reversed pimozide‐induced suppression of MYC expression, Buc: bucladesine, Pi: pimozide, *n* = 3. (R) Combined treatment: PRL reverses c‐MYC inhibitor (APTO‐253, 10058‐F4) mediated c‐MYC induction. (S) Therapeutic synergy: cell survival rates were measured after individual or combined treatment with the MDM2 inhibitor RG7112, PRL recombinant protein and c‐MYC inhibitors (APTO‐253, 10058‐F4), *n* = 5. Data expressed as mean ± SD unless specified; **p < .01, ***p < .001 by two‐tailed Student's *t*‐test; ns: not significant.

Given PRL's established role in liposarcoma and fibrosarcoma biology, we sought to delineate its mechanistic foundations through transcriptomic profiling. RNA‐seq analysis of SW872 cells treated with serum from hyperprolactinemic retroperitoneal liposarcoma patients (PRL‐6 h/12 h) versus healthy controls revealed distinct transcriptional landscapes via OPLS‐DA (Figure 7A and Table S6), As shown in volcano plot and heatmap: a total of 1176 DEGs were identified, comprising 546 up‐regulated and 630 down‐regulated genes (Figure 7B,C). KEGG pathway enrichment showed the significantly changed pathways include pathways in cancer, cytokine–cytokine receptor interaction, TNF signalling and IL‐17 signalling pathway (Figure 7D). Network analysis pinpointed MYC as the central node integrating multiple pathways (Figure 7E). Clinical validation via tissue microarrays confirmed elevated c‐MYC protein expression in liposarcoma versus adjacent adipose tissue (Figure 7F–L). Clinical relevance was confirmed using patient‐derived sera: preoperative RLPS serum induced higher c‐MYC expression versus post‐operative samples in SW872/HT1080 cells (Figures 7M,N and S24A). PRLR down‐regulation abolished PRL‐mediated c‐MYC induction (Figure 7O), confirming pathway dependency.

While the PRL–JAK–STAT axis is well characterised in tumours^22^ and JAK–STAT–c‐MYC signalling has been reported in leukaemia and glioblastoma,^23^ its role in sarcoma remains unexplored. To delineate PRL–JAK–STAT–MYC crosstalk, we analysed these effectors in PRLR‐knockdown and recombinant PRL‐treated cells. PRLR ablation down‐regulated c‐MYC, phosphorylated STAT3 and phosphorylated STAT5, whereas PRL treatment up‐regulated these mediators (Figures 7P and S24B). Preliminary results indicated that bucladesine up‐regulates PRL expression. We subsequently investigated whether bucladesine could rescue the STAT signalling pathway from inhibition mediated by the STAT5 inhibitor pimozide. Our findings demonstrate that pimozide suppresses STAT5 activity and down‐regulates MYC, whereas bucladesine effectively counteracts this inhibitory effect (Figures 7Q and S25). Subsequently, we treated SW872 cells with the c‐MYC inhibitors APTO‐235 and 10058‐F4. At the protein level, PRL was able to rescue the down‐regulation of c‐MYC induced by 10058‐F4 (Figure 7R). Additionally, APTO‐235 and 10058‐F4 enhanced the cytotoxic effects of RG7112, and PRL counteracted the combined cytotoxic effects of 10058‐F4 and RG7112 (Figures 7S and S26).

This multi‐platform evidence establishes a PRL–PRLR–c‐MYC signalling axis central to sarcoma proliferation and MDM2 inhibitor resistance in RPS pathophysiology.

## 4 DISCUSSION

This study identifies hyperprolactinaemia as a prevalent feature of retroperitoneal tumours (90%), spanning malignant sarcomas (well‐differentiated/dedifferentiated/pleomorphic/myxoid liposarcoma, leiomyosarcoma, fibrosarcoma, synovial sarcoma) and benign neoplasms (schwannoma/ganglioneuroma/desmoid fibromatosis). Mechanistically, we demonstrate SOX4‐mediated transcriptional regulation of PRL in liposarcoma. Cellular models revealed PRL‐driven activation of oncogenic JAK–STAT signalling and c‐MYC up‐regulation, coupled with enhanced proliferation and resistance to MDM2 inhibitors (e.g., RG7112). Given the diverse roles of PRL and our findings, PRL holds significant clinical potential for elucidating pathogenesis, improving disease management, refining treatment strategies, advancing prevention efforts and facilitating targeted drug development for RPS.

First and foremost, from a clinical perspective, we would like to discuss the following key points. PRL‐secreting tumours are implicated in diverse malignancies, including breast, prostate, lung, pancreatic, ovarian and endometrial cancers.^24^, ^25^, ^26^ We observed hyperprolactinaemia in 90% of patients with mesenchymal‐derived retroperitoneal tumours, while sarcomas originating from the trunk and extremities also derive from mesenchymal tissue, they are more readily detectable and manageable compared with retroperitoneal tumours which often develop insidiously and form a huge tumour. As shown in Figures 3F–K and S17A‐C, in the mouse model, the larger the tumour volume, the higher the prolactin level. As seen in Figure 3O and Figure S6, in the clinic, in liposarcomas with similar histological morphology, tumour volume is positively correlated with prolactin level. Sarcomas of the extremities and trunk are often detected and treated at a very small size and thus do not develop into huge tumours like retroperitoneal tumours, and therefore may not develop hyperprolactinaemia, although we have not conducted a comparative study. Early RPS detection is critical as tumours often grow large asymptomatically within the expansive retroperitoneum, encasing viscera; maximal initial resection is the cornerstone of management.^27^, ^28^ There is still a lack of strong evidence for prolactin as a biomarker for early diagnosis, and larger and more detailed cohort studies are still needed. It is worth noting that more than 50% of patients experience local recurrence despite R0/R1 resection. Reliable serum biomarkers for RPS diagnosis and monitoring are lacking. Our data show elevated PRL levels in primary and recurrent RPS, normalizing post‐operatively (Figure 2G). Although the evidence for PRL serving as a hallmark indicator of retroperitoneal tumours remains insufficient, it holds clinical significance as a monitoring marker for post‐operative recurrence in patients with retroperitoneal tumours presenting with hyperprolactinaemia.

Dopamine agonists, such as bromocriptine^29^ and cabergoline,^30^ are first‐line therapy for hyperprolactinaemia. Preclinically, bromocriptine significantly inhibited liposarcoma proliferation in a murine xenograft model (Figure S17), suggesting therapeutic repurposing potential. Novel targeted therapies specifically addressing the PRL–PRLR axis include neutralizing antibodies,^31^, ^32^ JAK2 inhibitors.^33^, ^34^ These findings collectively support exploring PRL–PRLR‐targeted strategies for RPS, which has limited therapeutic options. Hyperprolactinaemia suppresses the hypothalamic–pituitary–gonadal axis (GnRH, FSH, LH) and ovarian steroids, impairing folliculogenesis and causing anovulation.^35^, ^36^ Women present oligomenorrhoea, amenorrhoea, galactorrhea, headaches or visual deficits; men exhibit hypogonadism, decreased libido, erectile dysfunction or gynecomastia. Chronic hyperprolactinaemia associates with osteoporosis, cardiovascular morbidity, metabolic syndrome and increased breast/endometrial cancer risk.^37^, ^38^, ^39^, ^40^ Although we did not prospectively assess comorbidities in hyperprolactinemic RPS patients, these systemic sequelae necessitate prompt dopamine agonist therapy, especially in reproductive‐age patients, to mitigate gonadal dysfunction and preserve fertility.

Targeting MDM2, CDK4 and HMGA2 represents a promising strategy for RPS, particularly common well‐differentiated and dedifferentiated subtypes. MDM2 inhibitors include Nutlin derivatives, Milademetan, RG7112, Idasatulin, SP141 and SAR405838. It is well established that the principal mechanism by which MDM2 regulates tumour cells involves the negative regulation of wild‐type p53. As an E3 ubiquitin ligase, MDM2 inhibits the transcriptional activity of wild‐type p53 and facilitates its ubiquitination and degradation. Conversely, wild‐type p53 can up‐regulate the expression of MDM2, forming a regulatory feedback loop.^41^ Research on the role of prolactin in promoting drug resistance in conjunction with the loss of p53 also indicates that in sarcomas, PRL may be involved in the development of resistance to MDM2‐targeted therapies. MDM2 can also directly regulate molecules such as pRB, p107, E2F and HIF1‐α/VEGFA through a p53‐independent pathway.^42^ SW872 cells had previously demonstrated resistance to the MDM2 inhibitor SAR405838 (at a maximum concentration of 10 µM), while it was observed that higher MDM2 expression correlated with increased sensitivity to SAR405838.^43^ The resistance of SW872 may be attributed to the absence of MDM2 genomic amplification – a hallmark of well‐differentiated and dedifferentiated liposarcomas – along with relatively low MDM2 expression levels in SW872 cells, or potentially due to mutations in their p53 gene. However, in our study, when the concentration of SAR405838 was set at 20 µM, significant cell death occurred (Figure S13). Moreover, at the protein level, we observed MDM2 expression not only in SW872, 93T449 and 94T778 cell lines but also in HT1080 and fibrosarcoma‐derived cells – none of which exhibit MDM2 gene amplification – as well as in HCT116, QBC939, KYSE30, A549 and MB‐MDA‐231 cell lines (Figures 6A and S20). Compared with adjacent adipose tissue, MDM2 expression was predominantly up‐regulated in liposarcoma and fibrosarcoma tissues (Figure 6B). These findings indicate that MDM2 can be expressed even in the absence of genomic amplification, underscoring the broad therapeutic potential of targeting MDM2. However, multiple early‐phase clinical trials have failed to show a benefit with MDM2 pathway inhibition for DDLPS.^18^ The underlying reasons are multifactorial. Primarily, P53 mutations are observed across various sarcoma subtypes: osteosarcoma (80%), ewing sarcoma (10%), chondrosarcoma (20%), rhabdomyosarcoma (15%), leiomyosarcoma (50%), synovial sarcoma (10–20%), liposarcoma (10–20%), angiosarcoma (50%) and undifferentiated pleomorphic sarcoma (30%).^44^ Interestingly, when cells were treated with MDM2 inhibitors Nutlin‐3a and RG7112 or subjected to MDM2 knockdown, no significant changes were observed in p53 (mutated) in SW872 cells. In contrast, p53 (wild‐type) exhibited marked up‐regulation under the same experimental conditions (Figure S21). This phenomenon suggests that there is also a significant p53‐independent regulatory mechanism for MDM2 in SW872. In addition, substantial evidence has shown that MDM2 exhibits p53‐independent activities. These activities occur either via the ubiquitin–proteasome pathway (UPP)‐mediated degradation of target proteins or by competing with other E3 ubiquitin–protein ligases to prevent protein degradation, then effects that are involved in DNA repair, serine metabolism, cell proliferation and genomic instability.^45^, ^46^, ^47^, ^48^ Notably, a direct and bidirectional regulatory relationship exists between MDM2 and c‐MYC, which operates largely independently of p53 and collaboratively drives tumourigenesis.^49^ c‐MYC binds to the promoter region of the MDM2 gene, directly up‐regulating its transcription. Consequently, cells overexpressing c‐MYC exhibit elevated levels of MDM2.^50^ Similarly, MDM2 can regulate c‐MYC through p53‐independent mechanisms.^51^ In our study, prolactin enhances the chemoresistance of liposarcoma and fibrosarcoma models to MDM2 inhibitors (Figures 6Y,Z and S18, 19). We also demonstrate that PRL up‐regulates the expression of c‐MYC via the PRLR in liposarcoma and fibrosarcoma models (Figures 7 and S24). Clinically, patients with retroperitoneal liposarcoma exhibit hyperprolactinaemia in their serum, and high expression of c‐MYC is also detected in liposarcoma tissues. The c‐MYC expression in cells treated with preoperative serum from RPS patients is significantly higher than that in cells treated with post‐operative serum (Figures 7R and S24A). After treatment with a c‐MYC inhibitor, SW872 cells become more sensitive to MDM2 inhibitors (Figure 7S). These results indicate that c‐MYC plays a crucial role in PRL‐regulated chemoresistance of sarcoma cells to MDM2 inhibitors. Although this study has not elucidated the underlying mechanism, it provides a potential avenue for future research.

The c‐MYC proto‐oncogene regulates cell cycle progression, apoptosis, drug resistance and transformation, with dysregulation in ∼70% of human malignancies. Elevated c‐MYC may contribute to RPS features like recurrence, chemoresistance and poor immunotherapy response. c‐MYC also modulates the tumour microenvironment (TME), protecting proliferating cells from immune surveillance, influencing adaptive immunity, immune memory and tolerance, and inhibiting host anti‐tumour immunity, potentially explaining immunotherapy resistance. PRLR is ubiquitously expressed on immune cells (B cells, T cells, splenocytes, NK cells),^52^, ^53^ substantiating PRL's immunomodulatory role. Beyond endocrine functions, PRL acts as a pleiotropic cytokine secreted by immune cells, regulating lymphocyte activity paracrinely/autocrinely.^54^ RPS subtypes exhibit heterogeneous responses to PD‐1/PD‐L1 inhibitors, with DDLPS and WDLPS showing poor outcomes, linked to “cold tumour” features.^55^, ^56^ The potential association between PRL signalling and this resistance requires urgent investigation.

Furthermore, the PRL–PRLR axis significantly promotes sarcoma cell malignant proliferation (Figure 6). The endocrine function of adipocytes may thus be clinically significant for sarcoma recurrence. Sarcoma lesions within adipose tissue might exhibit accelerated proliferation and increased recurrence propensity due to a PRL‐enriched microenvironment, potentially explaining the 50–70% recurrence rate post‐resection. Consequently, complete adipose tissue excision during surgery may be critical for efficacy, considering the mitogenic effects of adipose‐derived PRL on residual cells.

Furthermore, from a biological perspective, the discussion will primarily focus on the following key aspects. Pituitary PRL biosynthesis is regulated by dopamine‐sensitive, Pit‐1‐dependent mechanisms via the proximal promoter.^57^ In contrast, extrapituitary PRL expression is controlled by a superdistal promoter located 5.8–9 kb upstream, which orchestrates tissue‐specific transcription independent of dopaminergic and oestrogenic signalling. This alternative promoter harbours conserved binding motifs for AP‐1, C/EBPβ and CREB transcription factors, facilitating cAMP‐responsive activation in decidual cells, lymphocytes and adipocytes.^58^, ^59^, ^60^ Furthermore, emerging evidence implicates STAT5A, oestrogen receptor β and thyrotropin‐releasing hormone as additional regulators of PRL gene expression.^61^, ^62^, ^63^ We identify SOX4 as a transcriptional regulator of PRL (Figure 5). We must also acknowledge a limitation of our study: although reporter assays and ChIP‐qPCR confirmed that SOX4 enhances PRL transcription, we did not perform direct validation of the transcription factor binding site by knockout of the distal SOX4 binding site in the PRL gene. This constitutes a methodological shortcoming in our research, aligning with genomic analyses showing SOX4 and TGFB2 up‐regulation in liposarcoma.^64^ Importantly, Huang et al. reported coordinated down‐regulation of PRL and IGFBP1 expression in SOX4‐deficient human endometrial stromal cells, corroborating our observations.^13^

PRL transiently up‐regulates during early preadipocyte differentiation, while PRLR expression is dynamically regulated throughout adipogenesis.^65^ Pharmacological analysis showed IBMX specifically induces PRL mRNA in preadipocytes, unlike INS, dexamethasone or PPARγ agonists (Figure 4F), consistent with cAMP‐mediated PRL induction involving PKA‐dependent and ‐independent pathways in eosinophils.^18^

It is established in clinical practice that dedifferentiated components of retroperitoneal liposarcoma can undergo transformation into well‐differentiated components, and conversely, well‐differentiated components may also transition toward a dedifferentiated phenotype.^66^ Notably, well‐differentiated liposarcoma exhibits considerable histological resemblance to adipose tissue. In our study, we observed a significant elevation in prolactin (PRL) levels during the adipogenic differentiation of MCS. Similarly, PRL expression was markedly increased in SW872, 94T778 and HT1080 cell lines upon activation of the cAMP–PKA signalling pathway (Figures 5L–P, S8 and S25). Based on these findings, we hypothesise that exposure of dedifferentiated sarcoma components to a cAMP‐activated microenvironment may promote their redifferentiation toward a well‐differentiated phenotype, analogous to adipogenic induction. This process would be accompanied by up‐regulated PRL expression, representing a transition from dedifferentiated to well‐differentiated status. Conversely, elevated prolactin may further stimulate malignant proliferation of dedifferentiated components, leading to an apparent increase in dedifferentiated elements that mimics a well‐to‐dedifferentiated transformation. Although detailed experimental data supporting this hypothesis are not provided in the current study, it offers a conceptual framework for investigating phenotypic plasticity in RPSs. This perspective on microenvironment‐mediated fate determination in liposarcoma aligns with the conclusions drawn from Nadège Gruel's research.^67^ We propose RPS pathogenesis may involve molecular aberrations during MSC differentiation, with PRL dynamics linked to adipocyte lineage commitment warranting investigation in sarcoma aetiology. Given the substantial tumour burden associated with large‐volume RPSs, the microenvironment‐induced hypothesis warrants further validation through analyses of key signalling pathways, single‐cell sequencing or organoid modelling across distinct tumour regions.

Next, we summarise the limitations of this study. (1) RPS exhibits significant histological heterogeneity. PRL expression patterns across subtypes and their shared pathological features require systematic study. PRL's diagnostic specificity and accuracy as a biomarker need rigorous validation. (2) Several critical questions regarding PRL‐mediated oncogenic signalling require resolution including the molecular mechanism of PRL‐induced c‐MYC up‐regulation in RPS; c‐MYC's pleiotropic functions beyond identified roles; bidirectional c‐MYC regulation of cellular homeostasis; PRL's immunomodulatory effects within the TME. (3) Although SW872 and HT1080 represent liposarcoma and fibrosarcoma models, respectively, they lack clear confirmation of retroperitoneal origin. These models may not fully capture the heterogeneity of RPS, necessitating further investigations across broader sarcoma subtypes. Additionally, more RPS‐derived cell lines need to be established and characterised. (4) Regarding the potential association between hyperprolactinaemia and sarcoma development in the trunk or extremities, it remains unclear whether PRL up‐regulation is specifically induced by the retroperitoneal microenvironment. Currently, clinical and experimental evidence on this subject is insufficient. (5) In our cohort of 100 RPS patients, 90% exhibited hyperprolactinaemia. However, confounding factors such as macroprolactin interference and renal/thyroid dysfunction require careful evaluation. Larger patient cohorts and comprehensive clinical data screening are warranted to derive more robust epidemiological insights. These findings establish a novel conceptual framework for understanding retroperitoneal tumour biology. However, addressing these unresolved questions through subsequent investigations will be crucial for translating these preliminary observations into clinically relevant applications.

## 5 CONCLUSION

**FIGURE 8:**
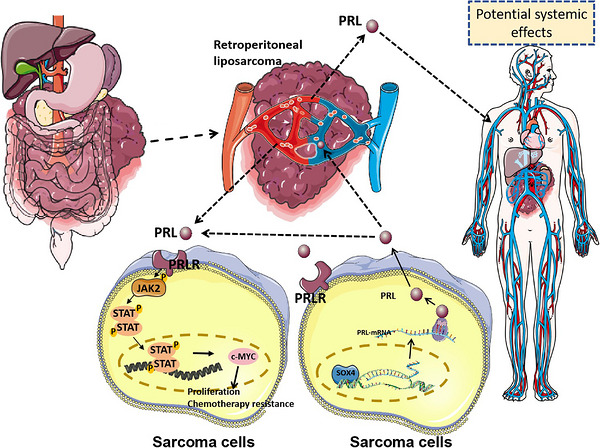
Proposed mechanistic model of PRL biosynthesis and oncogenic signalling in retroperitoneal sarcoma. Schematic representation delineates the transcriptional induction of PRL mRNA by SOX4 in retroperitoneal sarcoma cells. The PRL synthesised and secreted by these cells, enabling autocrine/paracrine binding to membrane‐associated PRLR. This ligand–receptor interaction triggers JAK–STAT phosphorylation cascade and up‐regulate c‐MYC proto‐oncogene, ultimately promoting the malignant proliferation of retroperitoneal sarcoma cells. Additionally, the PRL produced by these cells can enter the systemic circulation, potentially leading to widespread physiological effects.

We identified hyperprolactinaemia as a characteristic feature in many individuals with RPS. The increase in PRL primarily can occur through the SOX4–PRL. Elevated serum PRL stimulates RPS cells, activating JAK–STAT signalling and up‐regulating c‐MYC, promoting malignant proliferation in liposarcoma and fibrosarcoma. Beyond direct proliferation regulation, PRL may influence drug resistance, immunotherapy response and systemic effects (Figure 8). These discoveries enable novel therapeutic approaches targeting PRL blockade, offering valuable insights for RPS management. Targeting the PRL signalling axis – pharmacologically or via receptor inhibition – represents a strategic avenue to mitigate tumour progression and recurrence, warranting further exploration.

## AUTHOR CONTRIBUTIONS

WG. L., LL. L., HZ. W., CH. L. and FA. X. designed, supervised and funded the study. FA. X., LW. G., KR. Y., MM. X., S. W., GT. Y., RB. L., AB. Z., YH. C., YJ. N., Z. X. and WB. L. performed completed the experiments. LL. Q., B. Z., XG. X. and MM. X. collected and analysed clinical tissues. T. W. and CD. Y. analysed the data and writing‐reviewing and editing. FA. X. and LW. G. wrote the manuscript. All the authors have read and approved the article.

## CONFLICT OF INTEREST STATEMENT

The authors declare no conflicts of interest.

## ETHICS STATEMENT

All participants were enrolled and anonymised after approval by the institutional review board. Written informed consent was obtained from all participants except those who could not be contacted due to a lack of follow‐up. In these cases, permission was granted by the institutional review boards at each participating institution for the use of existing tissue samples for research. The present protocols were reviewed and approved by the Ethics Committees of all participating institutions, including Xiang'an Hospital of Xiamen University (No. XAHLL2021024, XAHLL2023004), Peking University People's Hospital (No. 2024PHB240‐001) and Peking University International Hospital (WA2020RW29). The protocols for the xenograft and metastasis experiments were approved by the Animal Ethics Committee of Xiamen University (approval No. XMULAC20210080).

## Supporting information



Supporting Information

Supporting Information

Supporting Information

Supporting Information

Supporting Information

Supporting Information

Supporting Information

Supporting Information

## Data Availability

Data collected for the study will be made available and shared to others though contact to the following address: lazyx@xmu.edu.cn, lwgang@xmu.edu.cn. The datasets supporting the conclusions of this article are provided within the article and its Supporting Information. Data of transcriptomic shifts during beige adipocyte differentiation from MSCs were obtain from Gene Expression Omnibus (GEO) DataSets (GSE125331). Data of sarcoma RNA‐seq were obtain from GEO DataSets (GSE71119).
