# Supplementary material for: Oncogenic driver and therapeutic target: Prolactin signalling axis in retroperitoneal sarcoma
Source: Clin Transl Med. 2026 May 5;16(5):e70669. doi: 10.1002/ctm2.70669 (PMC13139769; doi:10.1002/ctm2.70669)
Supplement: Supplementary file 8 — Supporting Information [file CTM2-16-e70669-s005.docx]

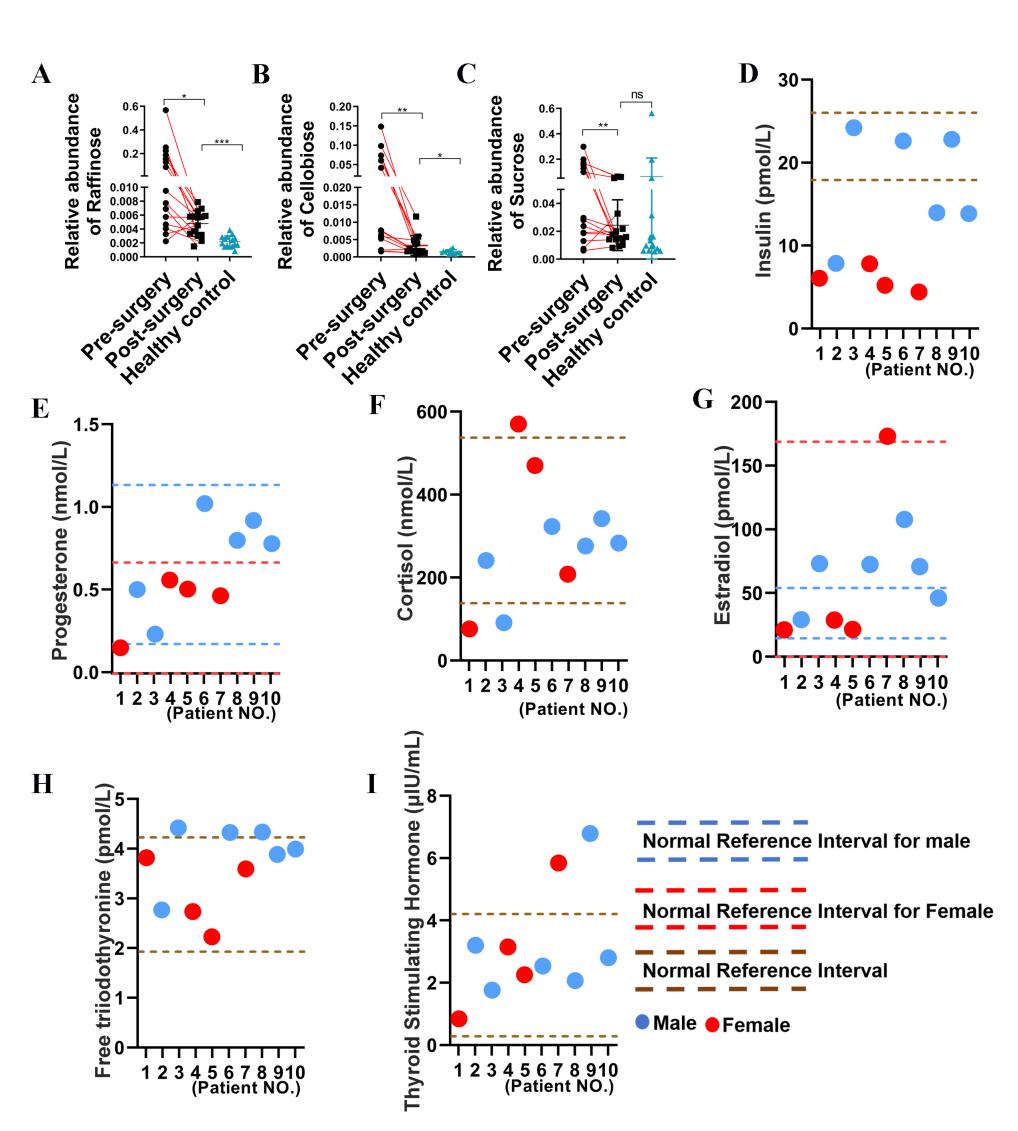


**Supplementary Figure 1.** Analysis of metabolites and hormone associated with galactose metabolism. (A-C) Mass spectrometry was employed to validate the relative concentrations of raffinose, cellobiose, and sucrose in the serum samples collected from the pre-surgery group, post-surgery group, and healthy subject group (n=15). (D-I) Concentration profiling of Insulin, progesterone, cortisol, estradiol, free triiodothyonine, and thyroid stimulation hormone in multi-omics discovery cohort. Data are presented as mean ± SD, n=10. **P < 0.01, *** P < 0.001; ns: Not significant.


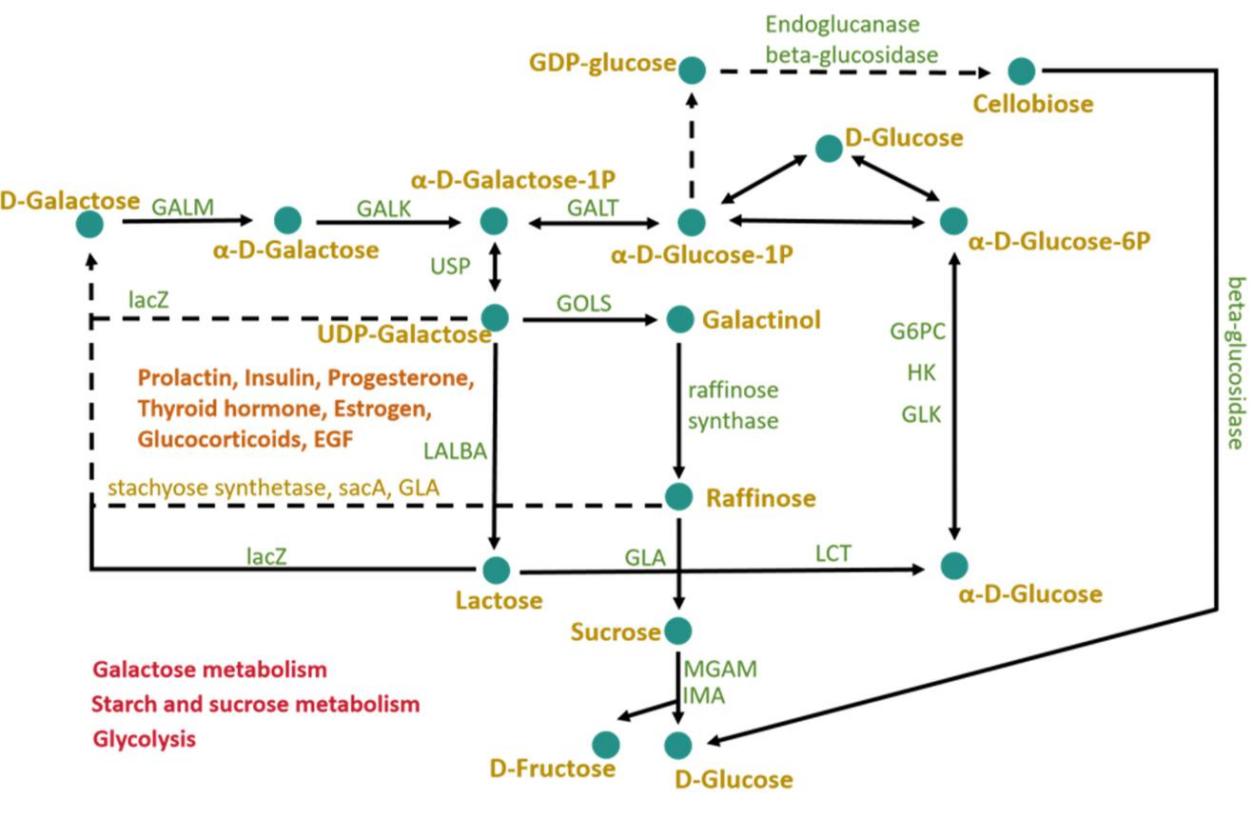


**Supplementary Figure 2.** Diagram of galactose metabolism,starch,and sucrose metabolism,and glycolysis pathways.


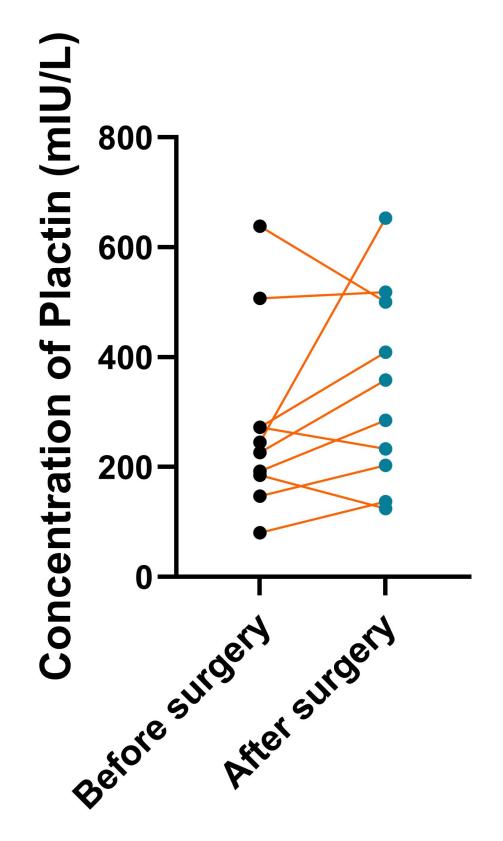


**Supplementary Figure 3.** Quantitative detection of prolactin in the serum of patients undergoing non-tumor surgery before and after operation，appendectomy (n=3), cholecystectomy (n=3), and gastric perforation (n=4) repair.


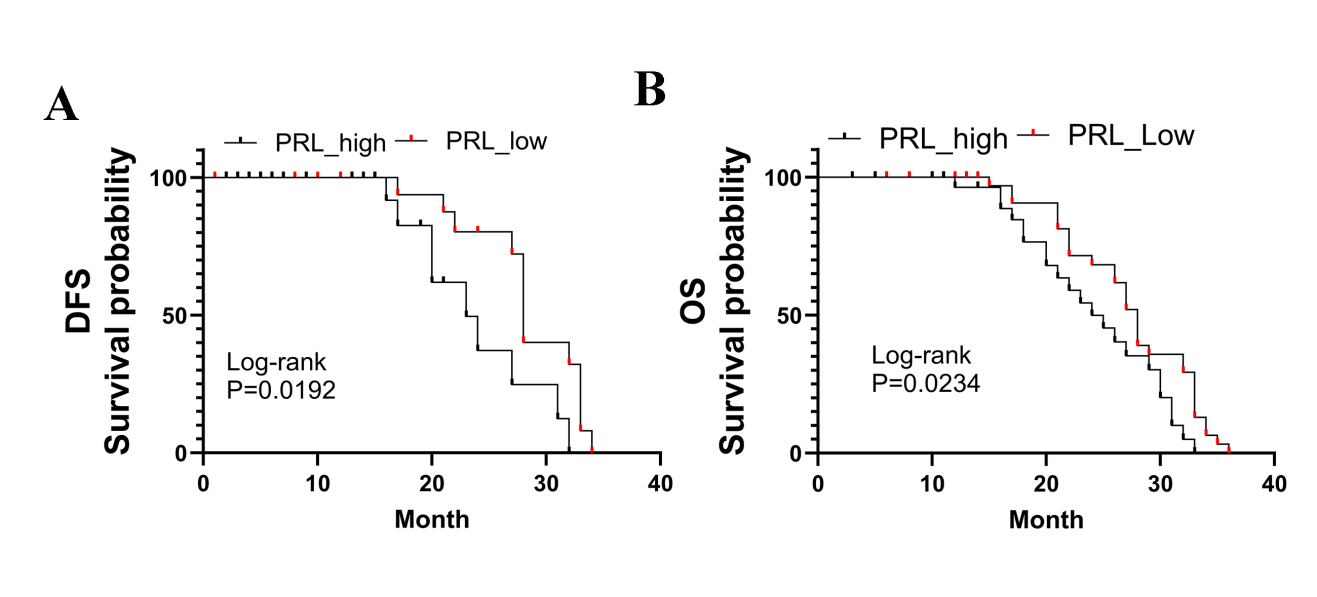


**Supplementary Figure 4.** Log-rank survival curves of DFS and OS in PRL-high (n=17) and PRL-low (n=18) group based on mRNA level (cutoff criteria: median).


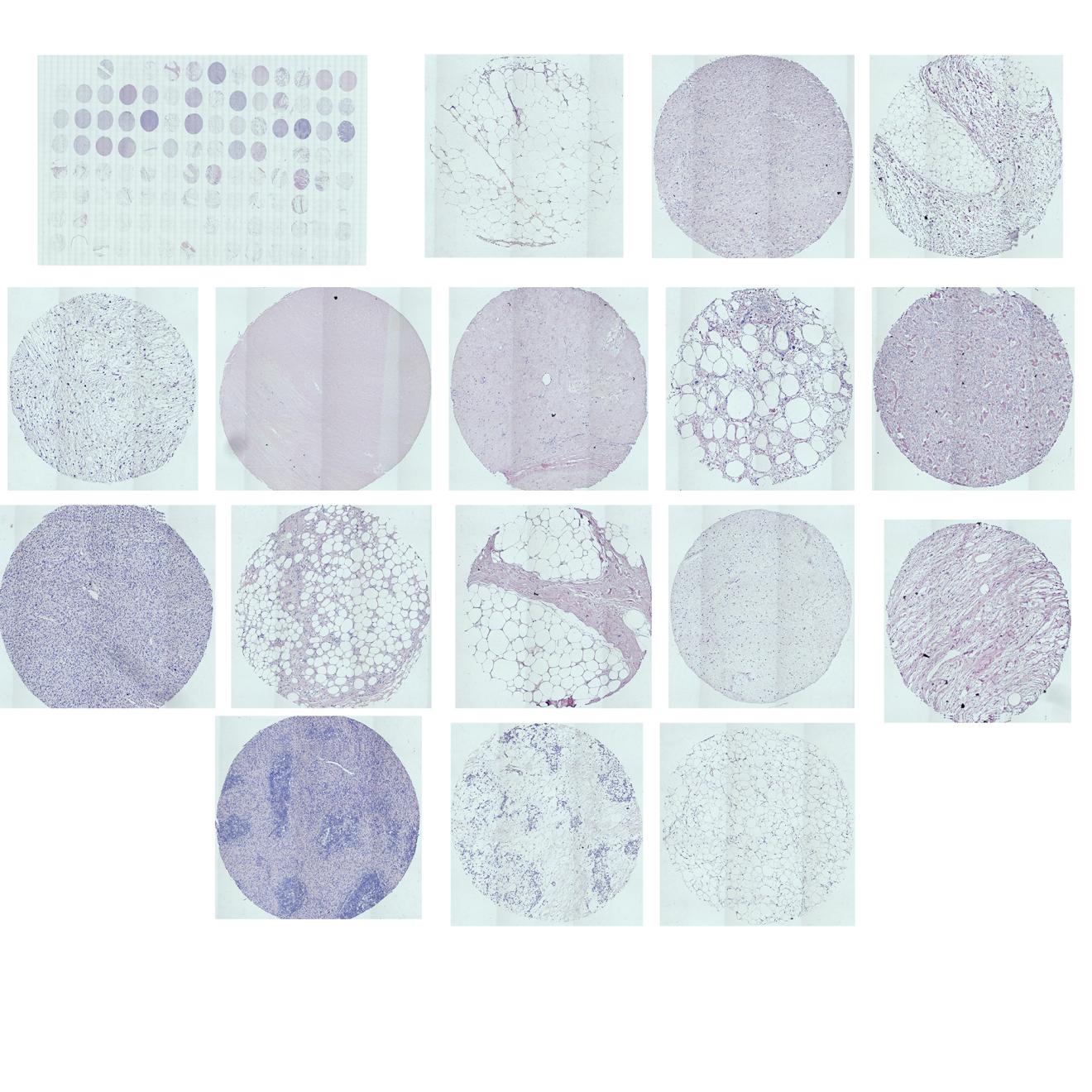


**Supplementary Figure 5** HE-stained clinical tissue sections of liposarcoma from different patients.


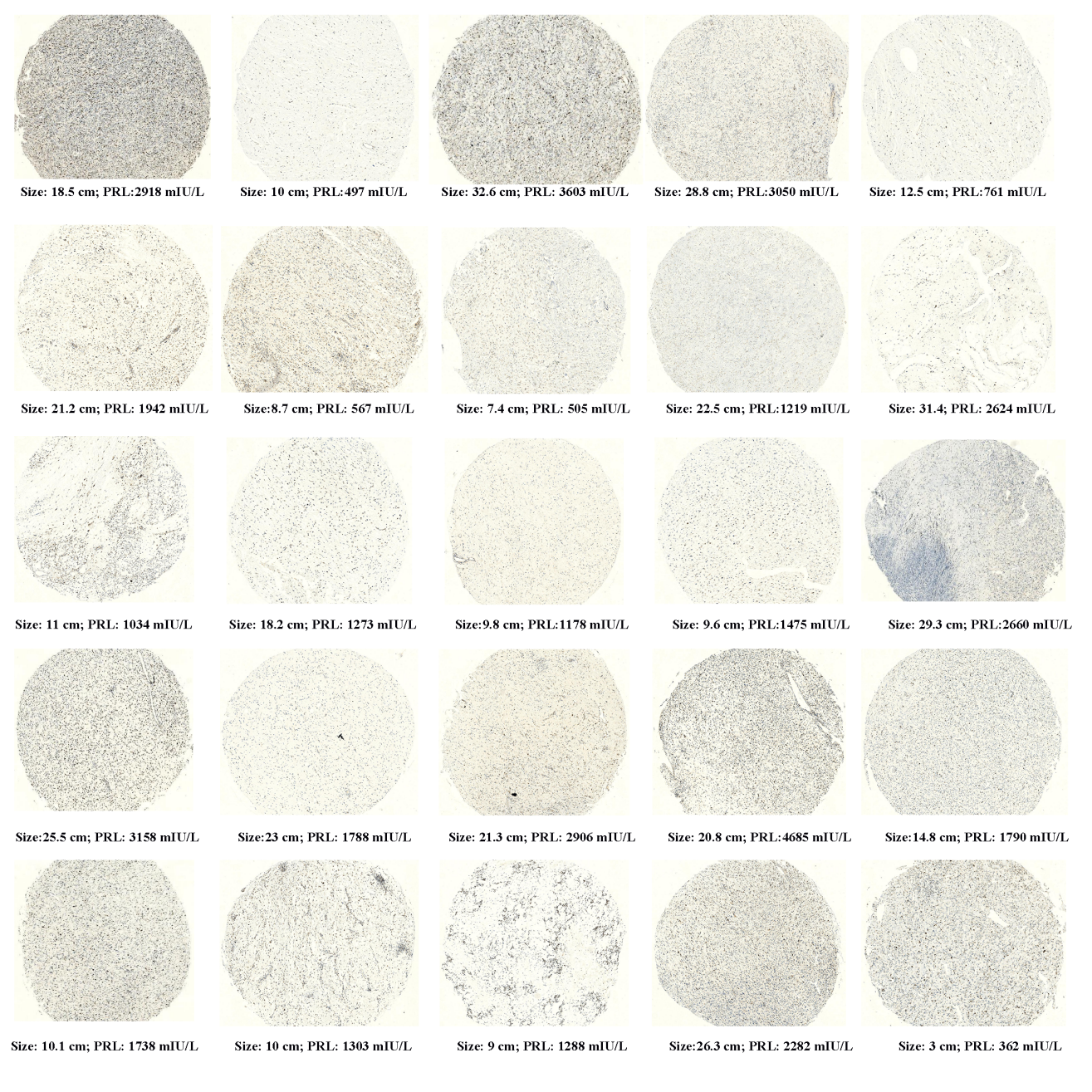


**Supplementary Figure 6** Selected immunohistochemical staining images of Ki-67 in retroperitoneal liposarcoma tissues for analyzing the correlation between prolactin levels and tumor size, along with corresponding patient prolactin levels and tumor dimensions （n=25）.


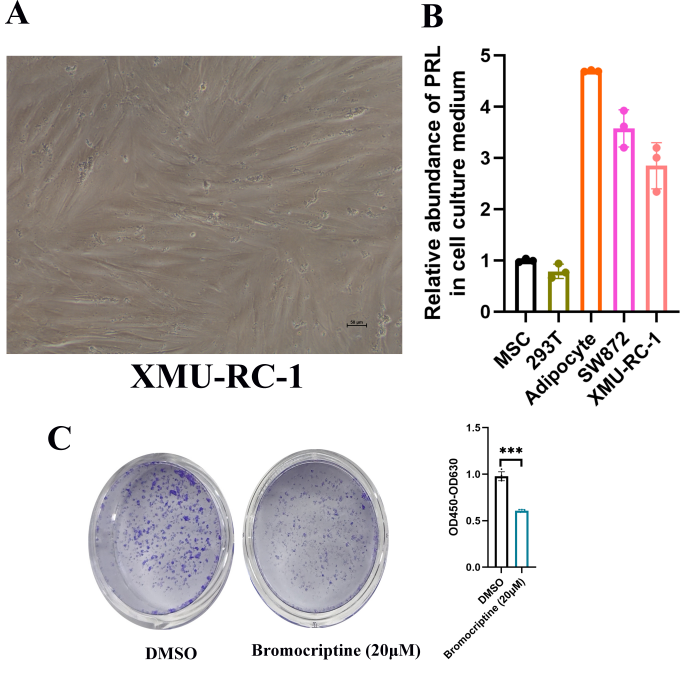


**Supplementary Figure** **7.**The XMU-RC-1 cell line derived from clinical liposarcoma tissue secretes prolactin (PRL). **(A)** Morphology of XMU-RC-1 under light microscopy. **(B)** ELISA analysis of prolactin secretion levels by XMU-RC-1 cells. **(C)**The proliferation activity of bromocriptine on XMU-RC-1 was assessed using the Cell Counting Kit-8 (CCK-8) method，n=3. Data are presented as mean ± SD, ***P < 0.001 by two-tailed Student's t-test.


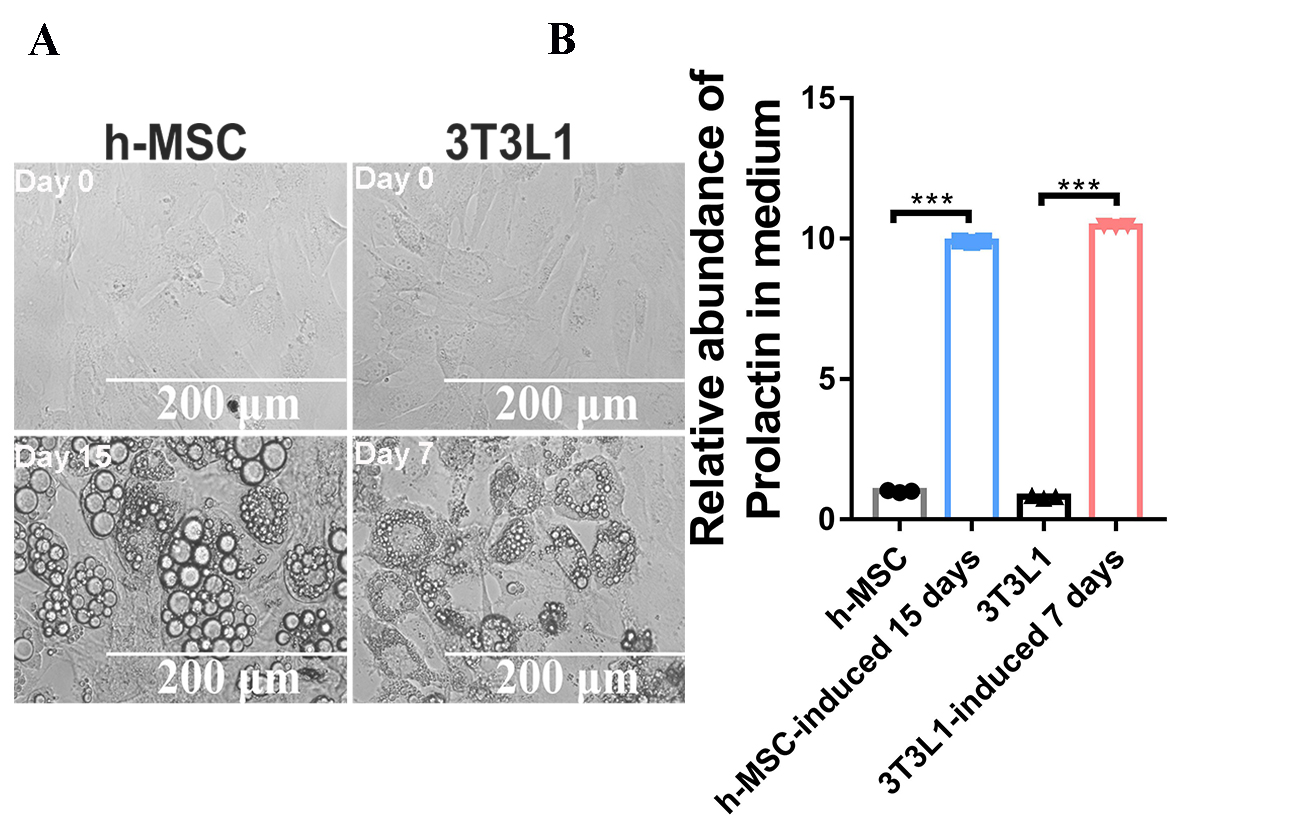


**Supplementary Figure** **8.** Comparison of prolactin expression before and after adipogenic induction in human mesenchymal stem cells and murine adipocyte precursor cells (3T3-L1), h-MSC: human mesenchymal stem cells. Data are presented as mean ± SD, n=3, ***P < 0.001 by two-tailed Student's t-test.

.


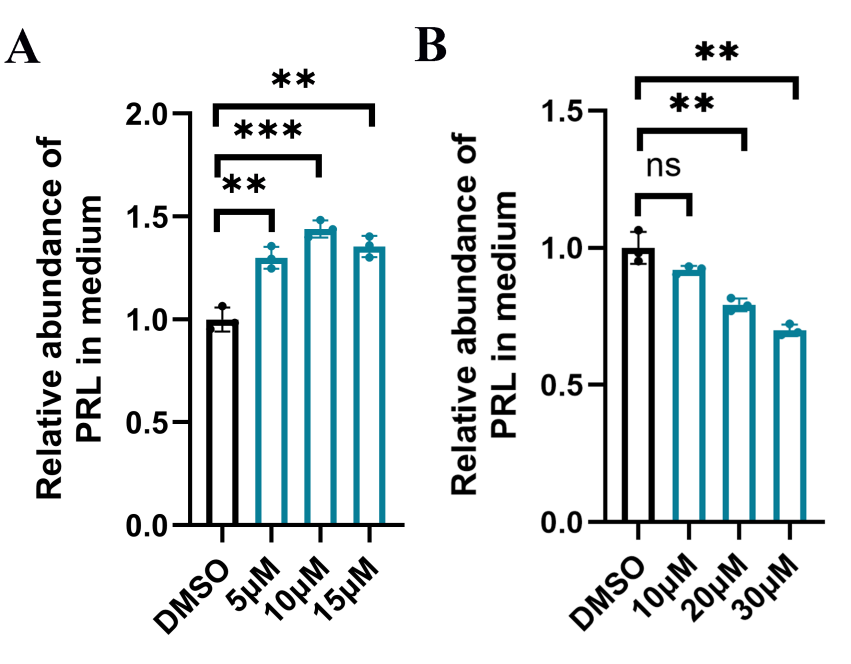


**Supplementary Figure 9.** Relative expression of PRL in the culture medium of SW872 cells treated with different concentrations of cAMP agonist Bucladesine (A) and dopamine receptor agonist Bromocriptine (B), n=3, **P < 0.01, ***P < 0.001 by two-tailed Student's t-test; ns: Not significant.


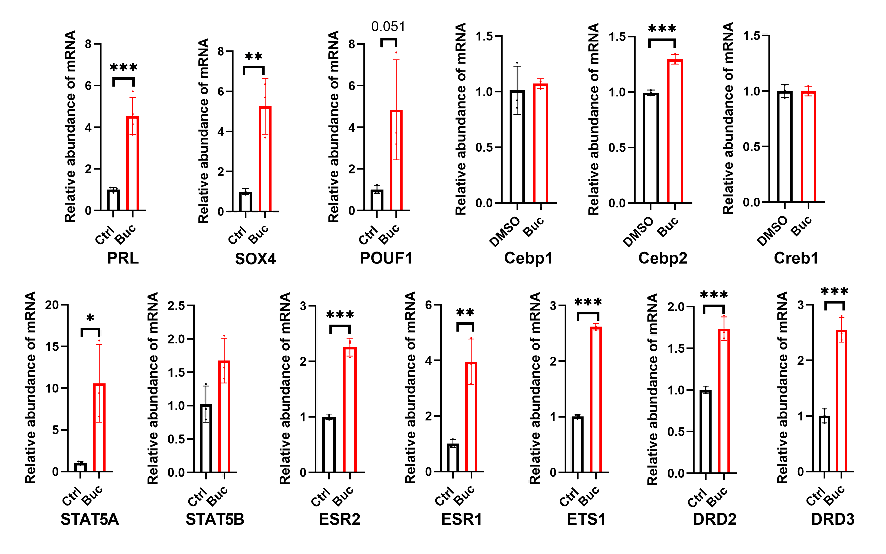


**Supplementary Figure 10.** qPCR detection of common transcription factors of PRL and dopamine receptor in SW872 cells treated with cAMP agonist Bucladesine (5μM), Buc: bucladesine, n = 3, *P < 0.05, **P < 0.01, ***P < 0.001 by two-tailed Student's t-test.


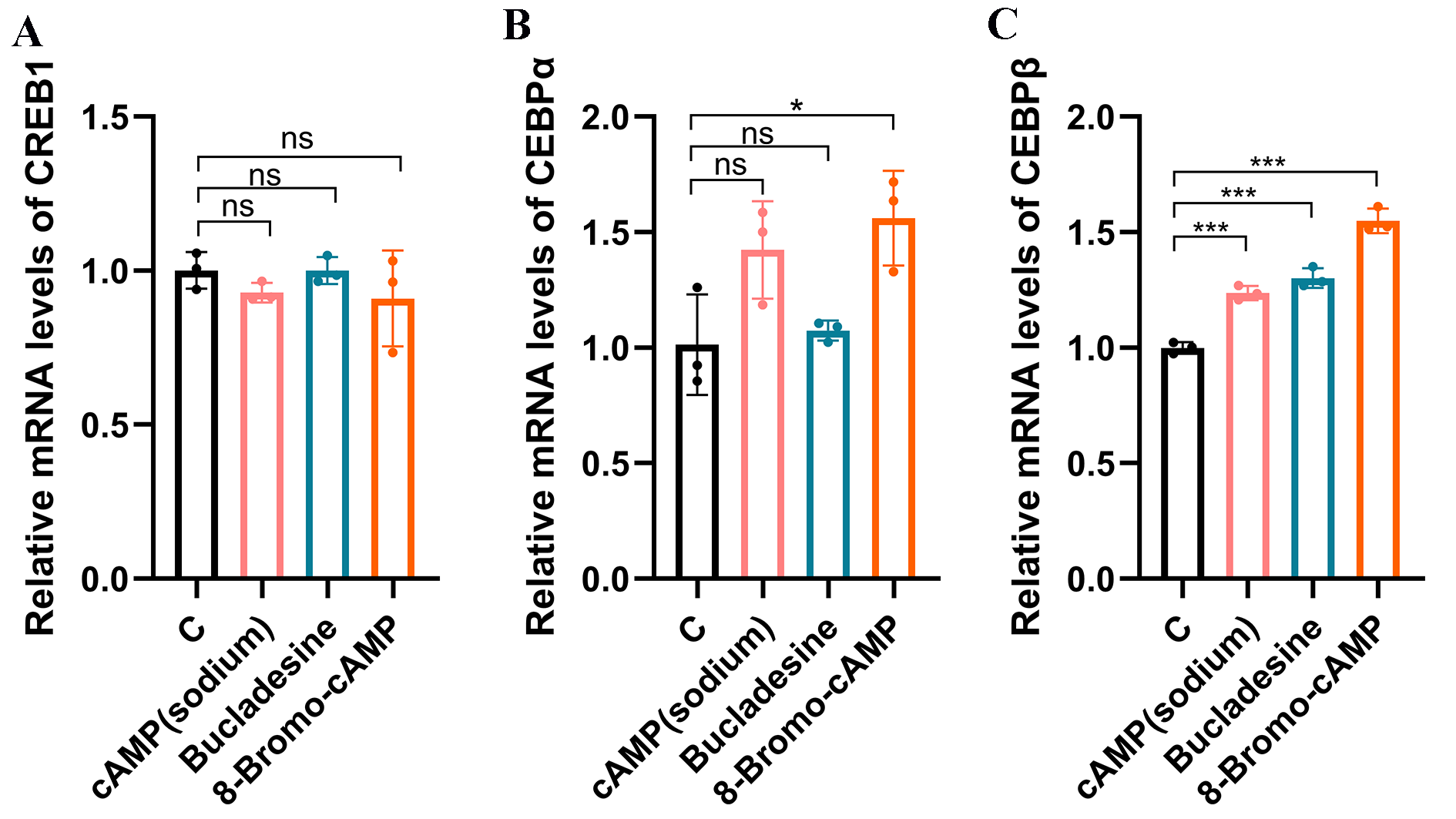


**Supplementary Figure 11.** Analysis of the mRNA level of CREB1 in response to cAMP and its analogs bucladesine and 8-Bromo-cAMP. Data are presented as mean ± SD, n=3, ns: Not significant.

.


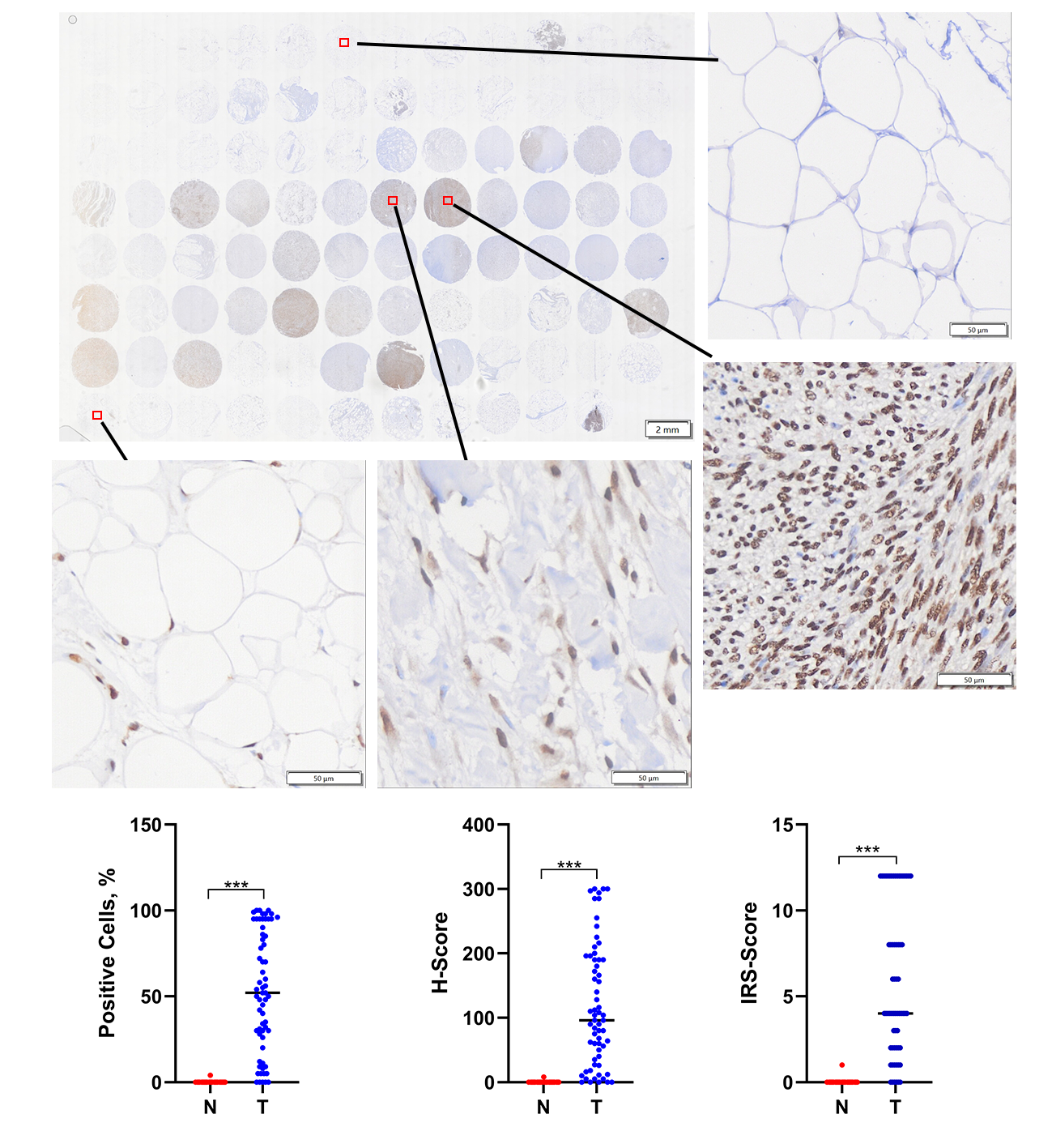


**Supplementary Figure** 12. IHC staining was performed on RLPS tissue and adipose tissue microarray using CREB1 antibodies. Positive cell% is calculated as the number of positive cells divided by the total number of cells; Histochemistry SCORE is calculated as ∑ (pi×i), where pi represents percentage intensity for weak/moderate/strong intensity levels (multiplied by 1/2/3 respectively). IRS is calculated as SI (positive intensity) multiplied by PP (positive cell ratio). Adipose tissue: n = 30; RWDLPS: n = 20; RDDLPS: n = 50. Data are presented as mean ± SD. ***P < 0.001.


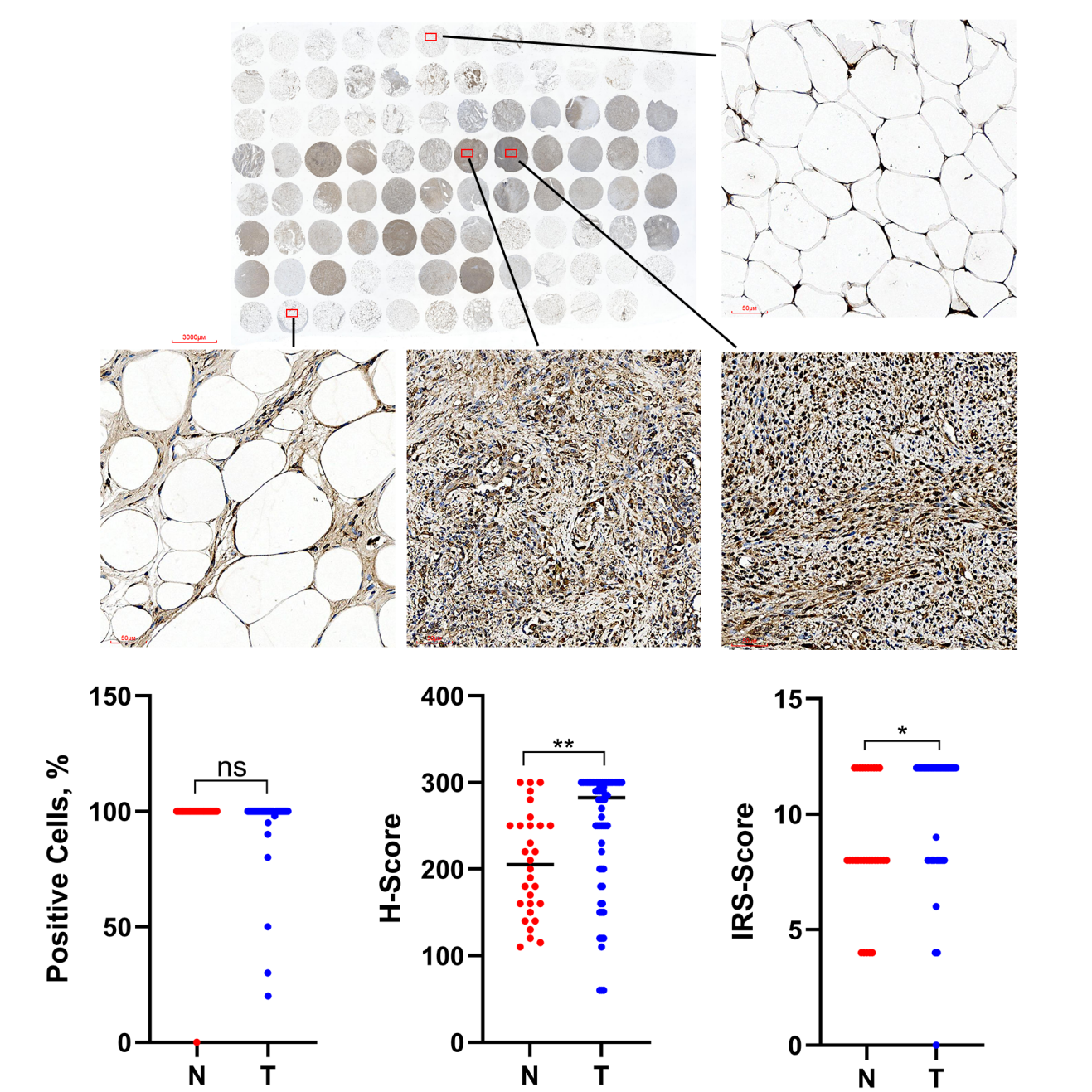


**Supplementary Figure 13.** IHC staining was performed on RLPS tissue and adipose tissue microarray using PRLR antibodies. Positive cell% is calculated as the number of positive cells divided by the total number of cells; Histochemistry SCORE is calculated as ∑ (pi×i), where pi represents percentage intensity for weak/moderate/strong intensity levels (multiplied by 1/2/3 respectively). IRS is calculated as SI (positive intensity) multiplied by PP (positive cell ratio). Adipose tissue: n = 30; RWDLPS: n = 20; RDDLPS: n = 50. Data are presented as mean ± SD. *P < 0.05, **P < 0.01, ns: Not significant.


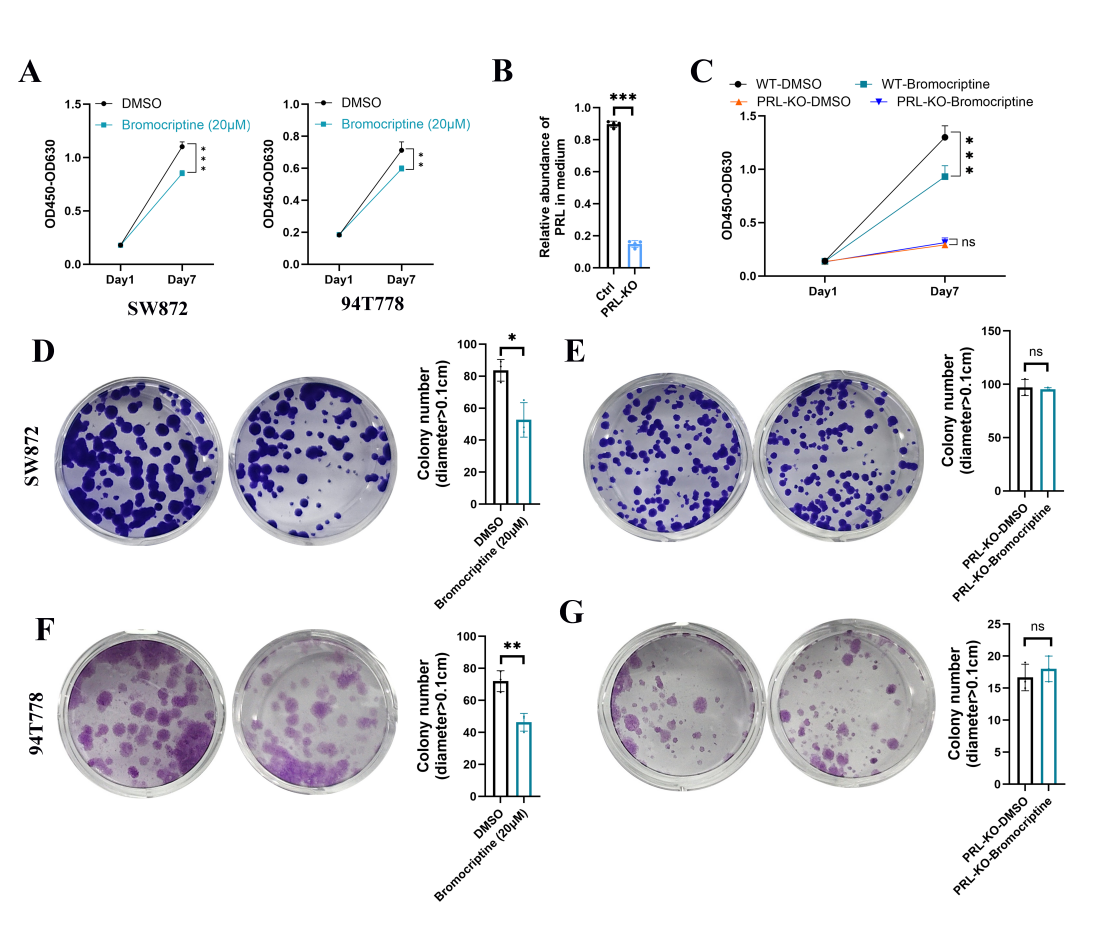


**Supplementary Figure 14.** Effects of Bromocriptine on Liposarcoma Cell Proliferation. (A) Bromocriptine significantly inhibited the malignant proliferation of liposarcoma cell lines SW872 and 94T778, n=5. (B) Knockout efficiency of PRL in 94T778 cells detected by ELISA, n=3. (C) CCK-8 assay revealed that bromocriptine markedly suppressed malignant proliferation in wild-type 94T778 cells; however, no significant inhibitory effect was observed in knockout (KO) cells, n=5. (D–G) Colony formation assay demonstrated that bromocriptine significantly inhibited malignant proliferation in wild-type 94T778 cells, whereas no notable suppression was detected in KO cells, n=3. Data are presented as mean ± SD. *P < 0.05, **P < 0.01, ***P < 0.001, ns: Not significant.


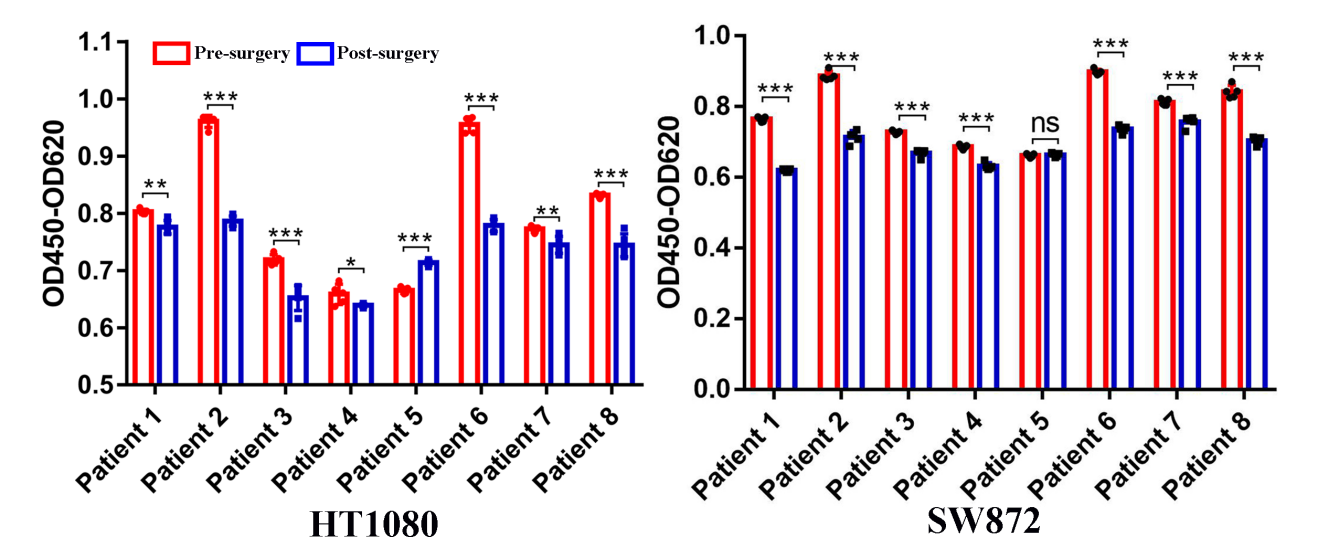


**Supplementary Figure 15.** Clinical serum bioactivity: CCK-8 proliferation index in cells treated with pre-/post-operative RLPS patient sera (10% v/v, n=8).Data are presented as mean ± SD. *P < 0.05, **P < 0.01, ***P < 0.001, ns: Not significant.


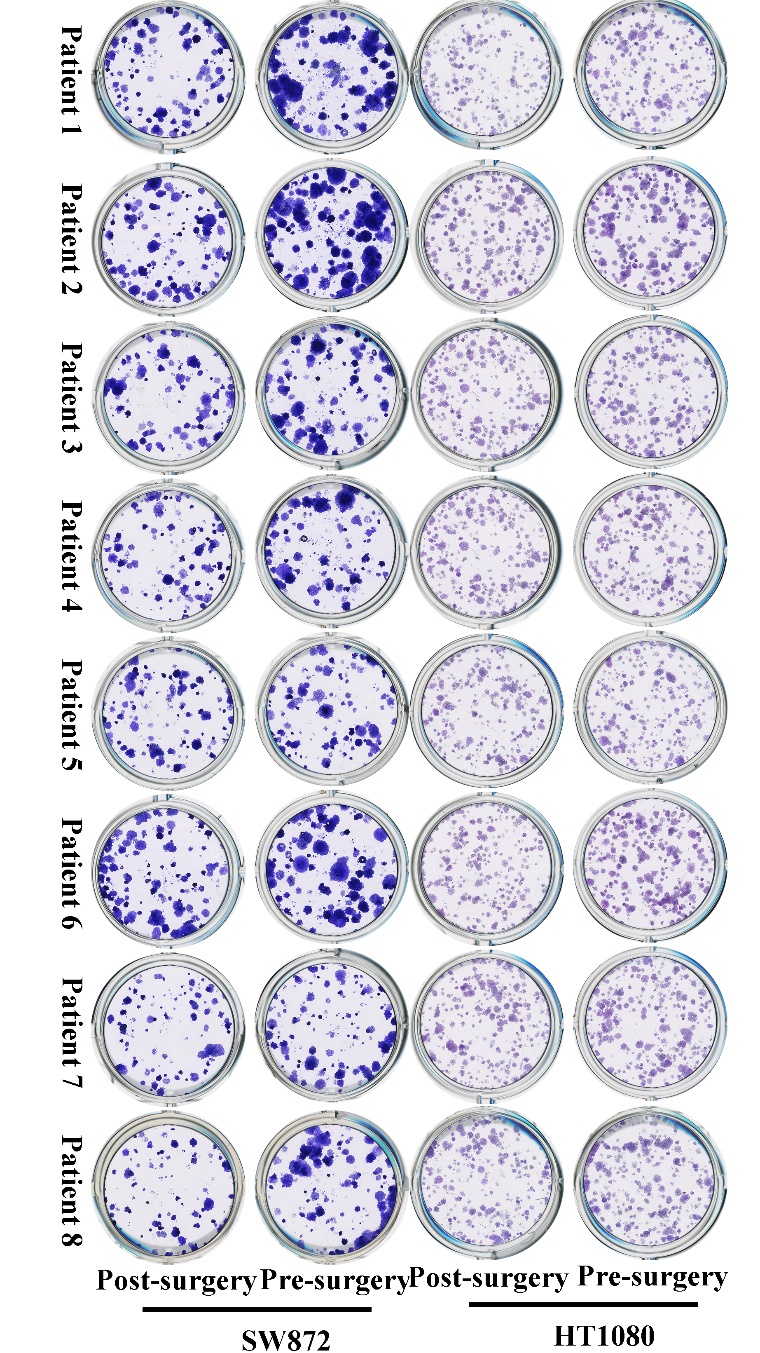


**Supplementary Figure 16.** Clinical serum bioactivity: Colony formation in cells treated with pre-/post-operative RLPS patient sera (10% v/v, n=8)


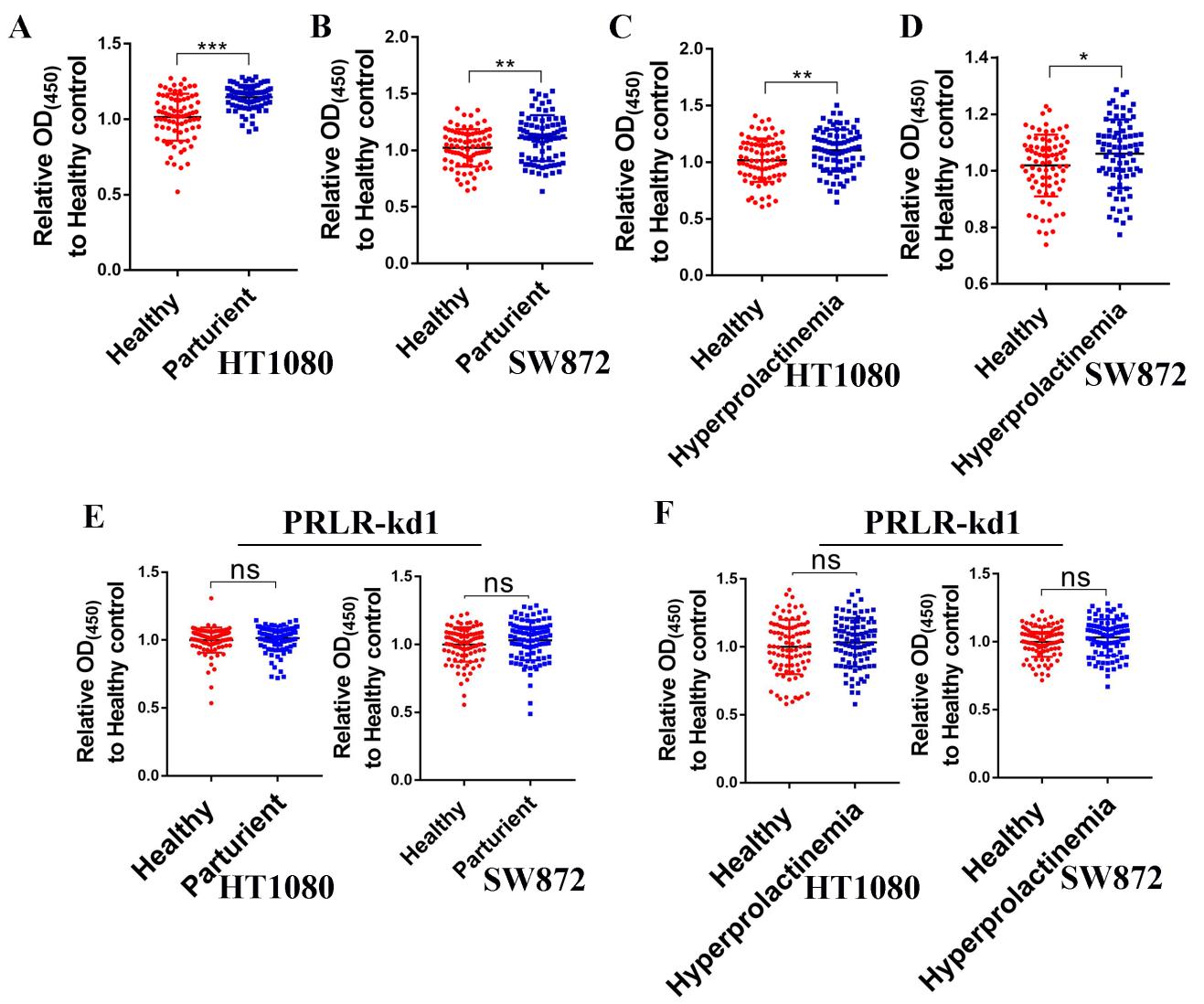


**Supplementary Figure 17.** The effect of serum from parturient and hyperprolactinemia patients on the proliferation of HT1080 and SW872. (A, B) The effects of serum from parturient women and healthy individuals on the proliferation of HT1080 and SW872 cells are investigated, n = 80. (C, D) The effects of serum from patients with hyperprolactinemia and healthy individuals on the proliferation of HT1080 and SW872 cells are studied (HT1080, n = 80; SW872, n = 79). (E) Effects of serum from parturient women and healthy controls on proliferation of HT1080 and SW872 cells after PRLR knockdown (n = 96). (F) Effects of serum from patients with hyperprolactinemia and healthy controls on the proliferation of HT1080 and SW872 cells after PRLR knockout (HT1080, n = 96; SW872, n = 92). Data are presented as mean ± SD. *P < 0.05, **P < 0.01, ***P < 0.001, ns: Not significant.


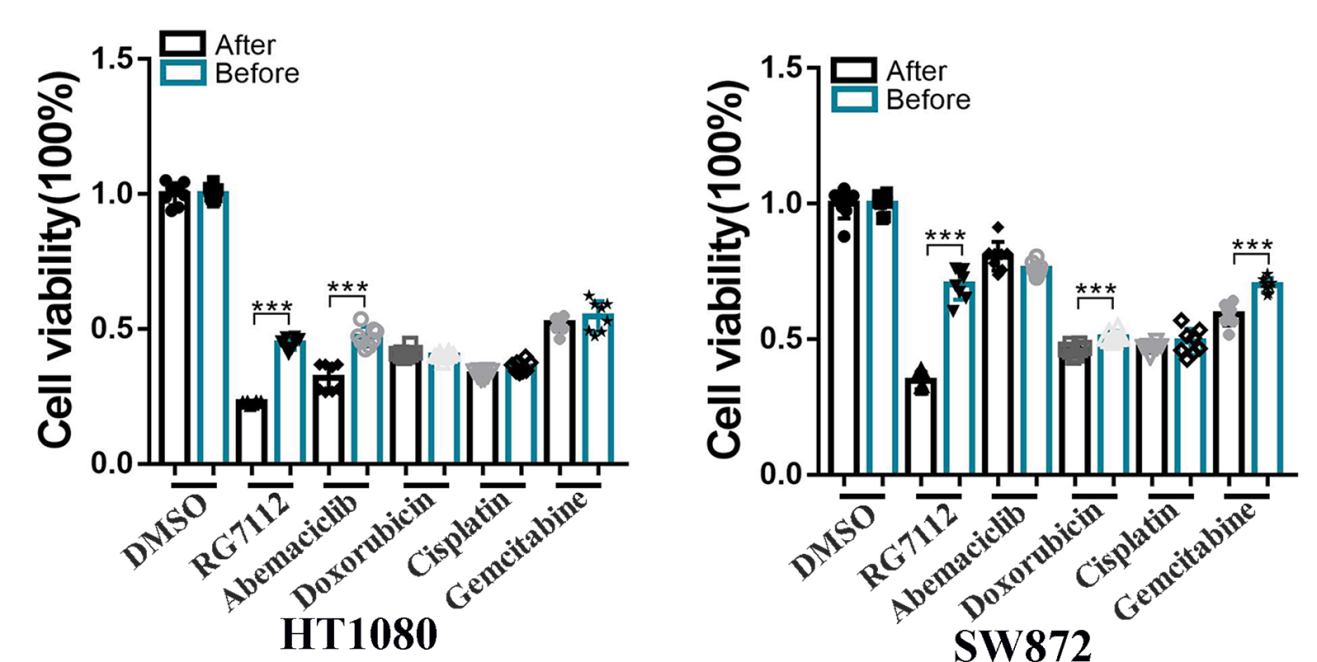


Supplementary Figure 18. Effects of pre- and post-operative serum from patients with RPS on regulating the sensitivity of HT1080 and SW872 cells to common chemotherapy drugs for RPS (n = 8).


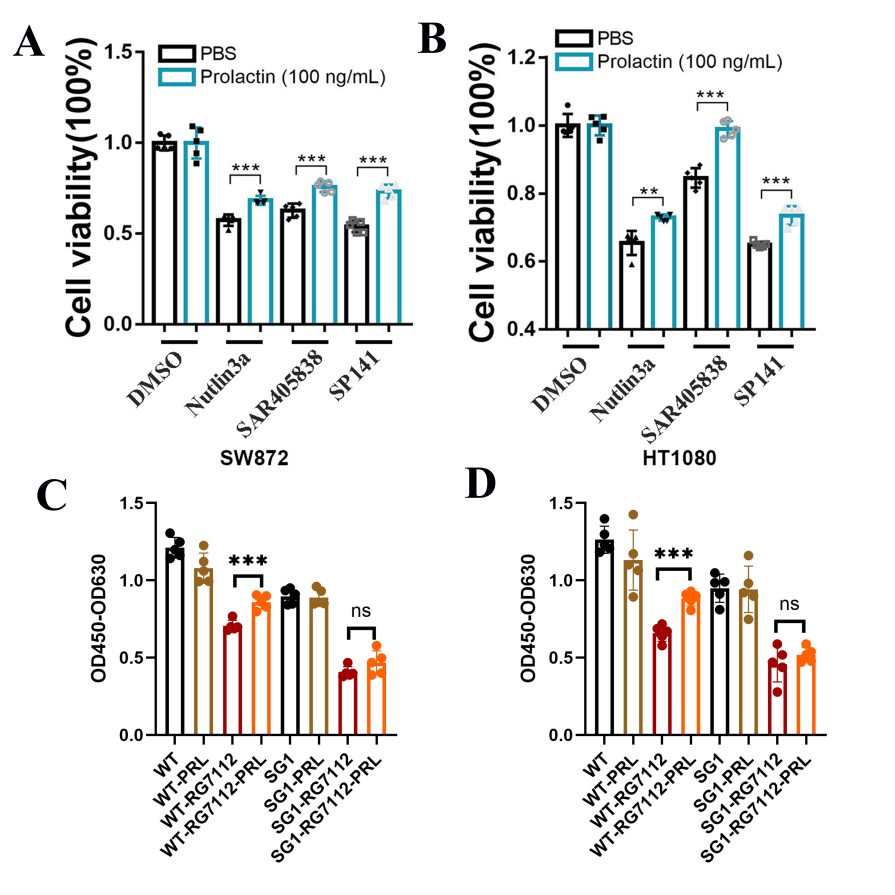


**Supplementary Figure 19.** Effects of PRL protein on regulating the sensitivity of SW872 and HT1080 to common inhibitors of MDM2.(A) and (B): Effects of PRL recombinant protein on the cytotoxicity of different MDM2 inhibitors. Nutlin-3a (25 μM), SAR405838 (20 μM), SP141 (1 μM), n = 5, treatment duration: 24 h. (C) and (D): Impact of PRL recombinant protein on the cytotoxicity of the MDM2 inhibitor RG7112 in PRLR-knockout cells. SG1: PRLR-sg1, RG7112: 20μM. Data are presented as mean ± SD. **P < 0.01, ***P < 0.001, ns: Not significant.


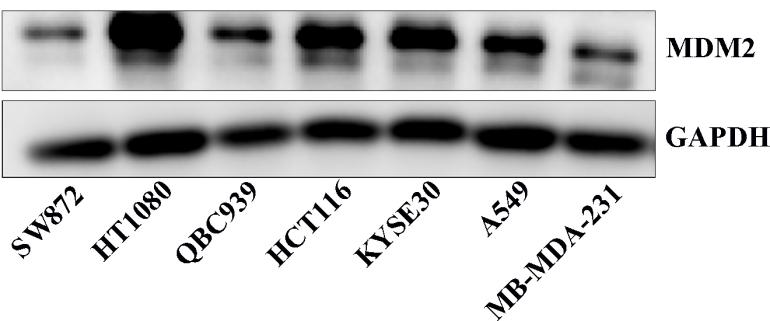


**Supplementary Figure 20** Western blot analysis was performed to detect MDM2 expression across various tumor cell lines, SW872: liposarcoma, HT1080: fibrosarcoma, QBC939: cholangiocarcinoma, HCT116: colorectal carcinoma, KYSE30: esophageal carcinoma, A549: lung adenocarcinoma, MDA-MB-231: breast cancer.


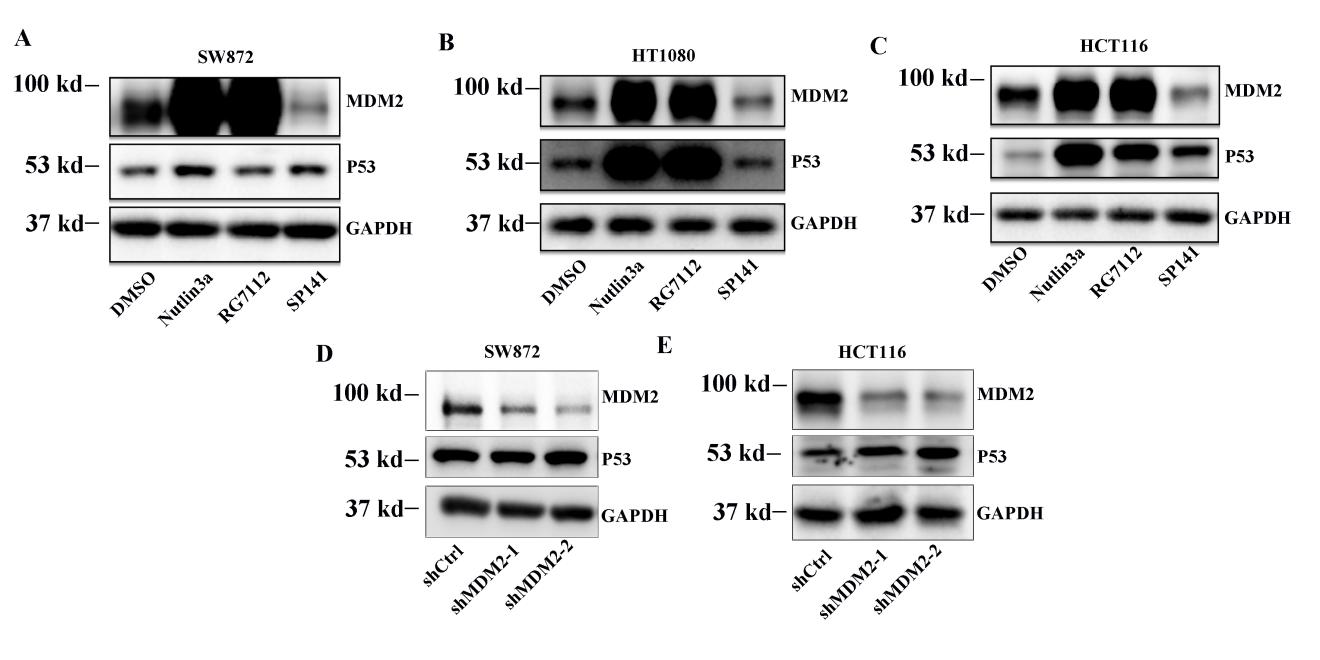


**Supplementary Figure 21** Western blot analysis was performed to detect alterations in MDM2 and p53 expression following MDM2 inhibitor treatment and MDM2 knockdown in SW872 liposarcoma, HT1080: fibrosarcoma cell, HCT116: colorectal carcinoma cell.


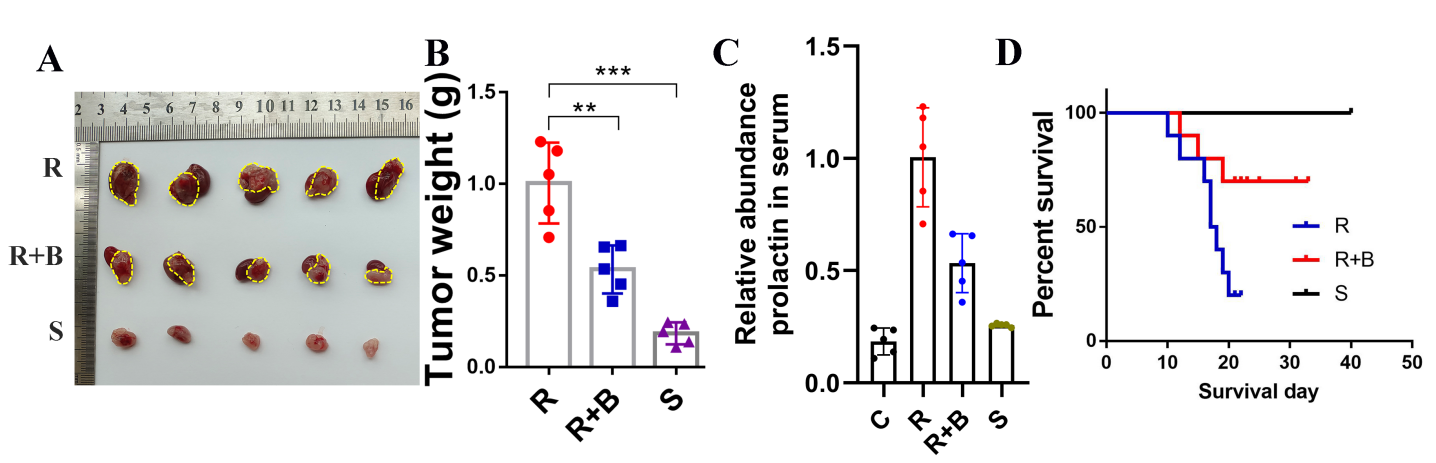


**Supplementary Figure 22.** Effects of bromocriptine on the proliferation of mouse fibrosarcoma WEHI164 cells in a retroperitoneal xenograft mouse model. (A, B)At the murine model level, bromocriptine effectively suppresses the malignant tumorigenic capacity of WEHI164 cells. Meanwhile, retroperitoneal inoculation demonstrates a significantly higher capacity for malignant proliferation compared to subcutaneous inoculation.， (n=5), R: retroperitoneal; B: Bromocriptine: 10 mg/kg, twice daily; S: subcutaneous inoculation. (C) Regulation of serum PRL levels in perirenal space-inoculated mice, subcutaneously inoculated mice, and bromocriptine-treated mice (n=5). (D) Survival statistics for perirenal space-inoculated mice, subcutaneously inoculated mice, and bromocriptine-treated mice (n=10). Data are presented as mean ± SD, *P < 0.05, **P < 0.01, ***P < 0.001, ns: Not significant.


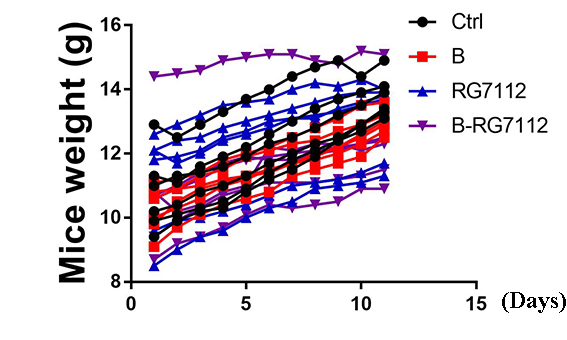


**Supplementary Figure 23**. The body weight of mice after treated with Bromocriptine and RG7112.


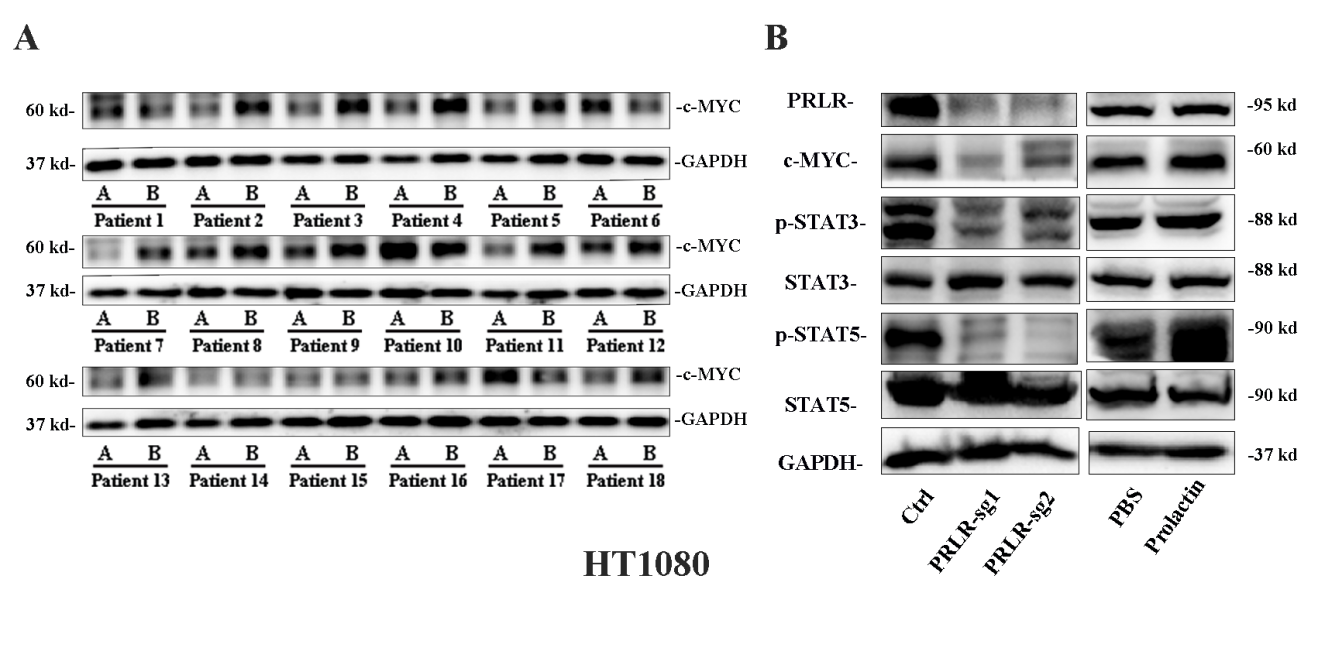


**Supplementary Figure 24.** (A) Western blot analysis of key proteins in the JAK-STAT signaling pathway were detected following downregulation of PRLR and addition of recombinant PRL (50ng/mL) in HT1080 cells. (B) Clinical correlation: pre-operative serum upregulates c-MYC vs post-operative serum in HT1080 cells.


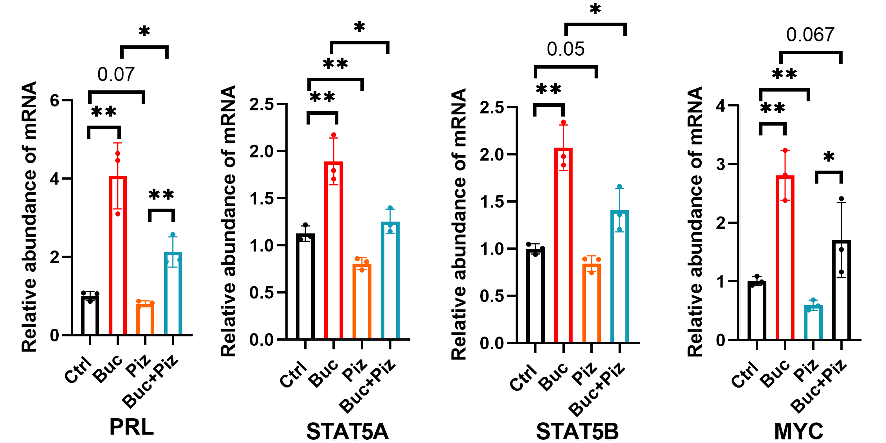


Supplementary Figure 25. Alterations in the expression levels of PRL, STAT5A, STAT5B, and MYC mRNA in HT1080 cells following combined treatment with bucladesine and pimozide, as determined by quantitative PCR analysis.


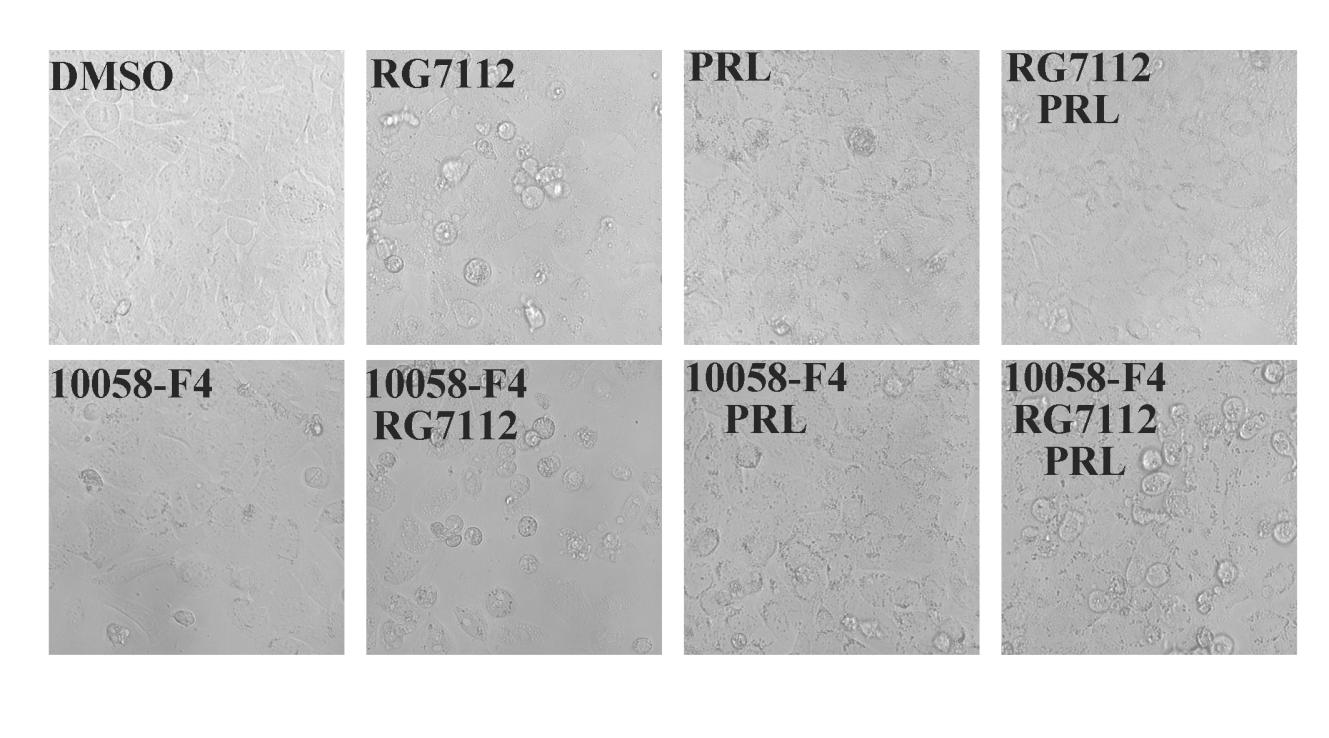


**Supplementary Figure 26.** Microscopic observation of SW872 cell status after individual or combined treatment with the MDM2 inhibitor RG7112, PRL recombinant protein, and c-MYC inhibitor, n=5.
